# Oral vaccination of wildlife using a vaccinia–rabies-glycoprotein recombinant virus vaccine (RABORAL V-RG^®^): a global review

**DOI:** 10.1186/s13567-017-0459-9

**Published:** 2017-09-22

**Authors:** Joanne Maki, Anne-Laure Guiot, Michel Aubert, Bernard Brochier, Florence Cliquet, Cathleen A. Hanlon, Roni King, Ernest H. Oertli, Charles E. Rupprecht, Caroline Schumacher, Dennis Slate, Boris Yakobson, Anne Wohlers, Emily W. Lankau

**Affiliations:** 1 Boehringer Ingelheim Animal Health, 1730 Olympic Drive, Athens, GA 30601 USA; 2 Conseils en Pharmacie et Biologie, Sainte Foy les, Lyon, France; 3 1088 chemin des Maures, 83440 Callian, France; 4 Institut Scientifique de Santé Publique, Service Maladies Virales, Laboratoire National de la rage, Direction Opérationnelle Maladies Transmissibles et Infectieuses, rue Engeland 642, 1180 Brussels, Belgium; 5 ANSES-Nancy Laboratory for Rabies and Wildlife, European Union Reference Laboratory for Rabies, WHO Collaborating Centre for Research and Management in Zoonoses Control, OIE Reference Laboratory for Rabies, European Union Reference Laboratory for Rabies Serology, Technopôle agricole et vétérinaire de Pixérécourt, B.P. 40009, 54220 Malzéville, France; 6 Centers for Disease Control and Prevention, Rabies Team Lead, Atlanta, GA 30333 USA; 7 Israel Nature and Parks Authority, 3 Am Ve’Olamo Street, Jerusalem, 95463 Israel; 8 Bertram, Texas USA; 9 The Wistar Institute, 3601 Spruce St, Philadelphia, PA 19104 USA; 10 Boehringer Ingelheim Animal Health, 29 Avenue Tony Garnier, 69007 Lyon, France; 11 USDA-Wildlife Services, 59 Chenell Dr, Concord, NH 03301 USA; 12 Rabies Department, Kimron Veterinary Institute, 20250 Bet Dagan, Israel; 13 LandCow Consulting, P.O. Box 5651, Madison, WI 53705 USA

## Abstract

RABORAL V-RG^®^ is an oral rabies vaccine bait that contains an attenuated (“modified-live”) recombinant vaccinia virus vector vaccine expressing the rabies virus glycoprotein gene (V-RG). Approximately 250 million doses have been distributed globally since 1987 without any reports of adverse reactions in wildlife or domestic animals since the first licensed recombinant oral rabies vaccine (ORV) was released into the environment to immunize wildlife populations against rabies. V-RG is genetically stable, is not detected in the oral cavity beyond 48 h after ingestion, is not shed by vaccinates into the environment, and has been tested for thermostability under a range of laboratory and field conditions. Safety of V-RG has been evaluated in over 50 vertebrate species, including non-human primates, with no adverse effects observed regardless of route or dose. Immunogenicity and efficacy have been demonstrated under laboratory and field conditions in multiple target species (including fox, raccoon, coyote, skunk, raccoon dog, and jackal). The liquid vaccine is packaged inside edible baits (i.e., RABORAL V-RG, the vaccine-bait product) which are distributed into wildlife habitats for consumption by target species. Field application of RABORAL V-RG has contributed to the elimination of wildlife rabies from three European countries (Belgium, France and Luxembourg) and of the dog/coyote rabies virus variant from the United States of America (USA). An oral rabies vaccination program in west-central Texas has essentially eliminated the gray fox rabies virus variant from Texas with the last case reported in a cow during 2009. A long-term ORV barrier program in the USA using RABORAL V-RG is preventing substantial geographic expansion of the raccoon rabies virus variant. RABORAL V-RG has also been used to control wildlife rabies in Israel for more than a decade. This paper: (1) reviews the development and historical use of RABORAL V-RG; (2) highlights wildlife rabies control programs using the vaccine in multiple species and countries; and (3) discusses current and future challenges faced by programs seeking to control or eliminate wildlife rabies.

**Electronic supplementary material:**

The online version of this article (doi:10.1186/s13567-017-0459-9) contains supplementary material, which is available to authorized users.

## Introduction

Globally, rabies is a neglected zoonotic disease of significant public health importance caused by enveloped single negative-stranded, negative-sense RNA viruses in the genus *Lyssavirus*, family *Rhabdoviridae*. Lyssaviruses are perpetuated by low level transmission within susceptible mammalian reservoir species populations, primarily meso-carnivores and bats. Currently, rabies virus is recognized as the most important lyssavirus species, given its high disease burden (i.e., mortality rate) among humans, domestic animals and wildlife. Rabies viruses cause acute, fatal encephalitis in mammals. Rabies is distributed widely on all continents except Antarctica and demonstrates both host species and geographic variation in viral genetics [1]. Preventing human rabies deaths requires a combination of approaches. The first steps to prevention are education about avoiding contact with suspect rabid animals, wound washing if exposure occurs, and provision of pre- and post-exposure rabies prophylaxis. Vaccination of domestic pets and livestock provides an added layer of protection. Finally, oral rabies vaccination (ORV) of wildlife limits and prevents the spread of rabies virus among terrestrial meso-carnivore populations and reduces risks of spill-over infections into domestic animal and human populations [2].

Prior to ORV development, wildlife rabies control measures consisted largely of eliminating or reducing reservoir wildlife populations through localized and targeted hunting, trapping, or poisoning [3]. However, these methods became controversial in some areas due to animal rights concerns and perceived negative impacts on biodiversity. Further, these approaches are labor intensive, may only control small-scale outbreaks, and in some instances were ecologically and economically questionable [4]. A more efficient and cost-effective wildlife rabies control strategy was needed.

Oral immunization of wildlife reservoirs was first considered as a potential approach to rabies control in the 1970s after genetic manipulation of rabies viruses under laboratory conditions yielded less virulent forms. Later biotechnology advances produced a recombinant vaccinia vector expressing the rabies virus glycoprotein gene [5]. An international collaboration of scientists leveraged these developments as they searched to find an efficient and cost-effective wildlife rabies control approach in the United States of America (USA) [6] and in Europe [7]. Early work focused on bait delivery to caged wildlife [8] and the first ORV field trial occurred in October 1978 in Switzerland using an attenuated rabies virus vaccine derived from the Street Alabama Dufferin (SAD) strain inserted in chicken head-baits [9]. Afterwards, large-scale ORV field trials targeting foxes were conducted in multiple European countries to control endemic fox rabies using a SAD-derived attenuated rabies vaccine (“standard” or SAD-B19 strain) [9].

Wide-spread environmental distribution of such attenuated rabies virus vaccines in oral baits, although effective, remains controversial in some countries. Some attenuated rabies virus vaccines retain residual pathogenicity for both non-target species, such as rodents and nonhuman primates, and target species (notably, striped skunk, *Mephitis mephitis*) [10–12]. Furthermore, attenuated rabies virus strains may retain pathogenicity for humans, posing a risk to those inadvertently contacting such vaccines. Thus, people exposed to SAD-derived attenuated vaccines or other attenuated rabies viruses should receive standard rabies post-exposure prophylaxis consisting of rabies immune globulin and vaccine [13].

Attenuated oral rabies vaccines for wildlife may also be limited in effectiveness due to the limited thermostability of RNA viruses [14] and inefficient or variable efficacy of oral immunization in some target species, notably major rabies reservoirs in North America, such as the raccoon (*Procyon lotor*) [15, 16] and striped skunk [10, 17]. Therefore, the global need for safer and more effective vaccines for ORV led to the development of the first recombinant candidate (a vaccinia–rabies recombinant vectored virus) licensed both in Europe and the USA to reduce the transmission of rabies virus within wildlife populations.

RABORAL V-RG^®^ (RABORAL V-RG^®^ is a registered trademark in the USA and elsewhere of Merial, Inc., which is now part of Boehringer Ingelheim) is one of two oral vaccine bait products recommended by the World Health Organization for wildlife rabies control.

RABORAL V-RG is a recombinant virus shown to be safe and effectives in reducing rabies virus transmission in wildlife [18]. RABORAL V-RG has been in continuous use since 1987 when it was first field tested in foxes in Belgium [19]. Thereafter, approximately 250 million doses have been distributed globally. This paper reviews the process development and biological properties of the V-RG vaccine, summarizes field experiences using RABORAL V-RG in multiple species and countries, and considers current and future challenges to successful use of ORV for wildlife rabies control and prevention.

## Main characteristics of RABORAL V-RG

### Development and characteristics

The vaccine construct (V-RG) used in RABORAL V-RG (the commercial vaccine-bait product) was developed jointly by the Wistar Institute, Philadelphia, USA and Transgene S.A., Strasbourg, France based on prior demonstration of foreign antigens being expressed in a vaccinia virus vector as a novel approach to vaccination [20]. Vaccinia virus (family *Poxviridae*) has been used for centuries as a vaccine to eradicate smallpox in humans [21]. Vaccinia virus was considered well-suited as a viral vector to create a recombinant ORV construct due to its thermostability, a large DNA genome capable of accepting additional foreign genes, the ability to elicit strong humoral and cell-mediated immune responses, the ability to grow to high titres in vitro, and an absence of oncogenic potential or evidence of viral integration into the host genome [22]. In addition, vaccinia virus is known to have a wide host range and yet no known wildlife reservoirs [21].

**Figure 1:**
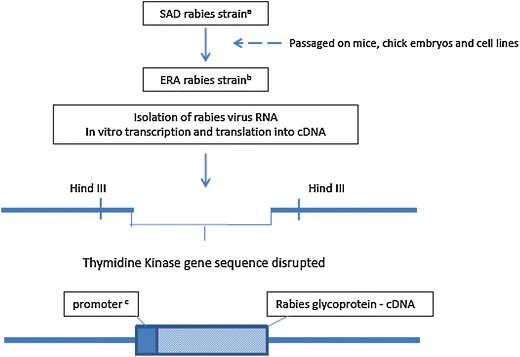
**Construction of the vaccinia–rabies glycoprotein recombinant vaccine (V-RG).** The SAD (Street Alabama Dufferin; Wandeler, 1991) rabies virus strain (**a**) was isolated from the salivary glands of a rabid dog in Alabama (USA) during 1935 and was attenuated to the ERA (Evelyn Rokitnicki Abelseth, 1964 [23]) rabies virus strain by repeated cell culture passages (**b**). The complementary DNA corresponding to the gene coding for the 524 amino acid G protein of rabies virus strain ERA was inserted into the double-stranded DNA genome of the vaccinia virus strain Copenhagen, under the control of (**c**) the 7.5 kDa vaccinia virus protein promoter [143], in the gene coding for thymidine kinase (TK). Shown is the TK region of the vaccinia virus genome with the inserted rabies virus G-cDNA from *Hind*III-digested plasmid pTG187-PRO.

The complementary DNA (cDNA) gene sequence coding for the 524 amino acid glycoprotein (G protein) of rabies virus strain ERA (Elizabeth Rokitnicki Abelseth; [23]) was inserted into the double-stranded DNA genome of a thermosensitive vaccinia virus strain Copenhagen (ts 26), under the control of the 7.5 kDa vaccinia protein promoter which interrupts the vaccinia thymidine kinase gene (TK) [20] (Figure 1). The G protein is the only viral protein present on the rabies virus surface and is well recognized by the mammalian immune system as a primary target for rabies virus neutralizing antibodies (RVNA) [24].

Modifications were made to the rabies G protein cDNA to ensure successful translation and antigen expression in the vaccinia virus vector. Site-directed mutagenesis was used to modify the rabies G protein cDNA sequence and then the modified cDNA was aligned with an early vaccinia virus promoter sequence inserted into a cloned copy of the non-essential vaccinia TK gene [20]. The resulting plasmid was transfected into vaccinia virus-infected cells. Double reciprocal recombination between the virus and the plasmid resulted in a recombinant attenuated vaccinia virus harbouring the rabies G cDNA [20]. The recombinant vaccine was called VVTGgRAB-26D3 [20] or V-RG [25]. A key advantage of this recombinant vaccine over attenuated rabies virus vaccines was the ability to trigger a strong immune response against rabies virus without the risk of the vaccine causing rabies.

### Initial laboratory safety and efficacy trials

Preliminary studies to assess the safety and efficacy of V-RG were performed in laboratory species (i.e., mice and rabbits). Vaccinia virus recombinants lacking TK functionality (i.e., TK-negative) were found to have decreased virulence in mice compared to wild-type virus without loss of immunogenicity [26]. V-RG was innocuous when administered to immunocompromised mice by the oral route and showed an expected decrease in virulence compared to the parental vaccinia strain on parenteral administration [27].

Administration of V-RG to mice by intradermal tail scarification or by footpad inoculation induced rapid production of RVNA, a strong specific secondary cytotoxic T lymphocyte response, and full protection against an intracerebral rabies virus challenge [25]. Administration of V-RG to rabbits by the intradermal, intramuscular, subcutaneous and oral routes at a dose of 10^7.8^ plaque forming units (PFU) induced RVNA production and protection from intracerebral rabies virus [28]. The minimum dose of V-RG shown to protect 50% of mice was 10^4^ plaque forming units (PFU) [28].

### Genetic stability

Genetic stability of V-RG was demonstrated in vitro after 10 passages in Vero cells and in a separate experiment after 11 passages in baby hamster kidney cells. After these passages, no change was found in the recombined region of the recombinant virus by restriction enzyme digest, electrophoresis and Southern blot analysis with a rabies G-protein gene probe and immunofluorescence (unpublished data, registration dossier/BL/AR DDD 128.91). In addition, efficacy of different passages of V-RG (5 and 10 passages on Vero cells) in laboratory mice vaccinated in the footpad was similar to that of the same vaccine prior to passage and vaccine sequences recovered after repeated passages in laboratory mice by multiple inoculation routes were genetically identical to the original V-RG construct (unpublished data, registration dossier BL/AR DDD 128.91).

Genetic stability and lack of reversion to virulence of V-RG was shown in vivo in red foxes (*Vulpes vulpes*) [29] and bank voles (*Myodes glareolus*) [11]. Voles were chosen for testing as a potential rodent reservoir for poxviruses in the environment [11]. V-RG was isolated from fox tonsils in the first 48 h after oral administration of V-RG to red foxes at a dose of 10^8^ tissue culture infective dose 50% (TCID_50_)/animal [29]. Vaccine isolated from fox tonsils 24 h after oral administration was then directly inoculated into other foxes to perform back passages. The vaccine was not isolated from the inoculated foxes and neither lesions nor clinical signs were observed during the 28-day observation period [29]. Back passage in bank voles by both the intracerebral and intradermal routes also demonstrated genetic stability. V-RG was not detected in bank vole tissues by culture on Vero cells after just a few passages and neither morbidity nor mortality attributable to the vaccine were observed in adult OF1 (Oncins France 1) mice inoculated with homogenates from each passage [11].

### RABORAL V-RG bait formats

**Figure 2:**
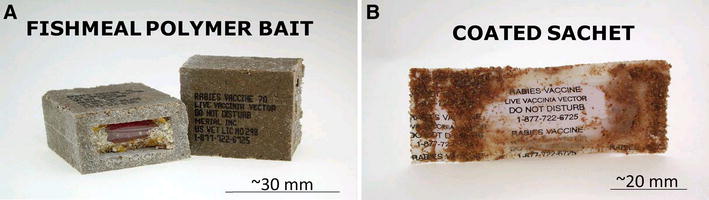
**Fishmeal polymer and coated sachet bait formats.** The fishmeal polymer (FMP) bait (**A**) is a cube made of extruded fishmeal and fish oil aggregated by use of a hydrophobic synthetic polymer (Bait Tek, Inc., Orange, TX). Wax is used to hold the vaccine-laden polyethylene sachet inside the bait. The coated sachet (**B**) is smaller and lighter than the FMP and consists of a vaccine-laden polyethylene sachet coated with wax, cod liver oil and fishmeal crumbles. Photo credit: Merial, Inc. stock photo image.

RABORAL V-RG is the commercial vaccine-bait product which consists of an edible bait-attractant coated plastic sachet containing a cell culture supernatant suspension of V-RG. RABORAL V-RG is currently available in two formats, with the vaccine-filled sachet either encased in a solid square fishmeal bait block (the fishmeal polymer block or FMP) or covered in a fishmeal-based crumble coating (the coated sachet; Figure 2). The FMP bait is an extruded mixture of fish meal and fish oil, aggregated by use of a hydrophobic synthetic polymer (Bait-Tek, Inc., Orange, TX, USA). Each sachet contains a minimum target fill volume of 1.5 mL at a minimum target titre of 10^7.7^ TCID_50_/mL, resulting in a typical delivered dose of approximately 10^8.0^ TCID_50_ per sachet. The vaccine-filled baits are distributed into wildlife habitats to induce immunity to rabies virus in target populations [30]. The FMP bait is primarily used for hand-baiting and bait stations in the USA, as well as controlling rabies in red foxes and golden jackals (*Canis aureus*) in Israel and raccoon dogs (*Nyctereutes procyonoides*) in South Korea. The product produced and registered in the USA is currently or was previously used as a licensed or an experimental wildlife ORV product in Canada [31], the USA [32], Israel [33], Ukraine [34] and South Korea [35]. The lighter-weight coated sachet bait is preferred for distribution over large geographic areas in the USA by aircraft (both helicopter and fixed-wing airplane) but is not exported.

Historically, different bait formats of RABORAL V-RG were produced to facilitate vaccine distribution and increase vaccine uptake by different target species. The original format used in Europe to target red foxes (*V. vulpes*) in the mid-nineties was a rectangular FMP block (52 mm × 33 mm × 21 mm) weighing approximately 40 g containing a plastic sachet affixed within the hollow of the bait by a lipid-based sealant (Rhone Merieux, Inc., Lyon, France) [36]. The European FMP product (which is no longer commercially available) had market authorizations in France, Belgium, Luxembourg and Poland.

To address the unique eating habits of key North American rabies reservoir species such as raccoons, a different shaped polymer bait and vaccine container was used experimentally during the early years of the USA program, consisting of a beeswax/paraffin ampule hand-filled with a target volume of 1.0 mL of vaccine surrounded by a cylindrical fishmeal polymer bait (Bait-Tek, Inc., Orange, TX, USA). The wax ampule bait was used for initial raccoon vaccination and non-target safety field trials [37, 38], but as the demand for raccoon doses increased in the USA and the manufacturer was asked to provide doses for coyotes (*Canis latrans*) and foxes, a large-scale automated production system was implemented using a new polyethylene plastic sachet primary container. The filled plastic sachet was folded and inserted into a square FMP bait casing (33 mm × 33 mm × 21 mm; weight approximately 23 g; Figure 2) that was lighter in weight than the French product and more efficient to pack into cartons for distribution than the cylinder bait. Field testing and laboratory evaluation of the square bait led to the manufacturer phasing out of the cylindrical wax ampule product. The square FMP bait product became the primary format sold from 1995 until 2005 (personal communication, J. Maki).

Further product development for the USA raccoon program led to the commercialization of a fishmeal powder-coated polyurethane sachet format (i.e., the coated sachet) made from an opaque white polyethylene plastic with a screen-printed label which was coated with wax, cod liver oil and fishmeal powder (Figure 2). The coated sachet (60 mm × 20 mm × 5 mm; weight approximately 9 g) was developed initially to facilitate vaccine uptake by juvenile raccoons [39]. The lighter design was intended to both make the bait easier for raccoons to manipulate and to reduce aerial distribution and production costs compared to the FMP [39]. During 1997 to 1998 the coated sachet was also field tested in Texas for use in coyotes. Initially, there was concern that coyotes might swallow the coated sachet baits intact due to this species’ tendency to gulp small food items. However, coyotes effectively punctured the vaccine container during ingestion [39]. The coated sachet was found to be attractive to raccoons and coyotes and was as effective as the FMP baits for inducing immunity against rabies virus [39, 40]. The coated sachet became the primary product format used in the USA for raccoons, coyotes and foxes as of 2005.

### Tetracycline biomarker

The FMP bait contains tetracycline hydrochloride (150 mg/bait) as a calciphilic biomarker that deposits in the growing bones and teeth of animals consuming the baits [41]. Teeth (canine or premolar) or jaw bone samples can be analysed to detect tetracycline residues as fluorescent lines in the bone matrix under ultraviolet light microscopy [42].

Several factors influence the quality of tetracycline biomarker test results, including sample type (i.e., bone, tooth) or location, laboratory protocols, and animal age [42, 43]. Tetracycline marking in raccoon canine teeth is often more efficient than in first or second premolars, for example, as much as 1.6 times [31], but canine teeth cannot be extracted humanely from a live animal in a field setting. Even premolar collection is considered a relatively invasive procedure requiring field anaesthesia. Additionally, laboratory protocol parameters may impact results, such as the plane of the tooth section (i.e., transverse versus longitudinal), the use of a mounting medium and the thickness of the sections [43]. Animal age may also influence results as tetracycline residues may be diluted in younger animal tissues due to bone growth and remodelling [42, 43].

Tetracycline marking has been an important ancillary measure for ORV program monitoring both in Europe and North America. It has been used to gain understanding of bait exposure and uptake rates in target and non-target species, particularly in early placebo environmental safety studies [44]. A benefit of tetracycline as a biomarker is that the dose and frequency of bait exposure can be observed in a single tooth sample. Animals consuming multiple baits during a single campaign receive a higher dose of tetracycline and have increased intensity of tetracycline residue lines. If ingestion of multiple baits occurs over a season or multiple years, a single tooth specimen may show multiple distinct tetracycline rings [42]. Thus, tetracycline marking has also proved valuable for estimating yearly primo-vaccination rates during multi-year programs and for assessing frequency of re-vaccination (i.e. ingestion of 1 or more booster dose) [45].

Decades of biomarker evaluation in ORV programs, however, have established that tetracycline marking and RVNA serologic results are not always congruent. Whereas tetracycline is ingested and absorbed to deposit efficiently in bone (i.e., the bait is consumed), oral vaccination occurs in lymphoid tissues in the oropharynx after the vaccine sachet is punctured and vaccine enters the oral cavity. Thus, animals that swallow without chewing the vaccine container or those that puncture the container but discard the bait may have conflicting results on biomarker and serologic assays. Tetracycline in baits may degrade over time or convert to a chemical form that is a less effective marker when ingested [41]. Further, long-term use of tetracycline for environmental release may contribute to consequences such as ecotoxicity or antimicrobial resistance development [14]. Thus, tetracycline biomarker is a potentially useful tool for ORV campaign testing and development. However, continued use of this biomarker as a monitoring tool should be evaluated in context of other measures of program success, such as post-baiting seroprevalence in target animals sampled in vaccinated areas and rabies case trends over time, and potential environmental impacts of extended use [14].

### Resistance and thermostability of RABORAL V-RG

A key feature of RABORAL V-RG is its higher thermostability when compared to other attenuated oral rabies vaccines. In vitro, V-RG is highly stable under refrigeration temperatures (4 to 7 °C), with a minimal decrease in titre (reduction of 10^0.4^ TCID_50_/mL) after 18 months of storage at 4 °C [46], which facilitates storage and distribution conditions at refrigerated but not freezing temperatures.

V-RG is also broadly stable at operational temperatures as confirmed by laboratory and field experiments. In the laboratory, vaccine titre declined by 10^1.3^ TCID_50_/mL after 56 days at 20 °C and by 10^1.5^ TCID_50_ after 7 days at 37 °C [46]. Under field conditions, V-RG vaccine remained stable in FMP baits over a period of 1 month, despite large variations in environmental temperatures (−20 to 22 °C) and several natural freezing and thawing cycles recorded during the trial [46]. The 1-month stability testing period was to include an anticipated delay in bait uptake in the field since foxes are likely to cache food [47]. Vaccine stability experiments were also conducted in France with FMP baits during summer months (July and August). Environmental temperatures in areas with shade in this study ranged between 8 and 37 °C and sun-exposed areas reached 57 °C. When vaccine baits were placed on grass under shade conditions for 3 weeks the average titre loss was 10^0.8^ TCID_50_/mL. Exposure of baits to the sun on barren ground for 3 weeks resulted in a titre loss of 10^2.2^ TCID_50_/mL, while remaining attractive to foxes even after exposure to these warm conditions [48].

The stability of V-RG in FMP baits manufactured in the USA was evaluated in New Jersey (Cape May) during early ORV campaigns [49]. Vaccine-filled wax ampules or wax ampules within FMP baits were placed within the vaccination area and exposed to sunlight and varying climatic elements for 10 months (May–February of the subsequent year). Environmental temperatures ranged between −14 and 36 °C during this study. The V-RG vaccine contained in ampules was protected from the environment by the FMP bait and remained stable during the first 3 months and did not substantially decline until mid-winter, 8 months later. In unprotected wax ampules, virus titre declined gradually and virus was undetectable after 3 months of sunlight and warm temperatures [49]. For coated sachets tested under field conditions, the mean titre loss after 4 weeks in shade (under forest canopy) was 10^0.9^ TCID_50_ compared to a loss of 10^3.3^ TCID_50_ for baits placed in forest edge or open field environments [50]. Raccoons removed 64 to 83% of baits within the first week of distribution in early USA field trial use [49, 51]. Thus, significant vaccine titre loss is not expected in the field during the critical period of expected bait uptake by wildlife, enabling use of this vaccine-bait product under a broad range of field conditions to access different target species as well as enact emergency campaigns to address an outbreak regardless of season.

## V-RG vaccine safety

### Vaccine vector primary multiplication site, excretion, and dissemination

Following consumption of a vaccine-bait, V-RG replicates locally in the oropharynx at the site of vaccine exposure. The virus has comparable tissue tropism of the parental vaccinia strain and is not typically disseminated systemically or excreted in oral secretions for more than a few days after administration, significantly reducing the likelihood of environmental shedding. V-RG was detected up to 48 h post-inoculation in the tonsillar tissue by virus isolation and in tonsils, buccal mucosa and soft palate of foxes using polymerase chain reaction (PCR) following administration of 10^8.0^ TCID_50_ via the oral route [29]. Due to its limited replication the vaccine was not detected in any organ, brain, blood, salivary glands (parotid and maxillary glands) or faeces by PCR [29]. Similarly, in orally vaccinated raccoons (10^7.8^ PFU/animal), V-RG was recovered from buccal mucosa, tonsils and submandibular/parotid lymph nodes during the first 48 h post-administration but no viremia could be detected during the 14 days post-inoculation [52]. The vaccine was not detected by virus isolation from faeces and saliva swabs of squirrel monkeys and chimpanzees inoculated intradermally by scarification (10^8.0^ PFU/animal) or by the oral route (10^9.0^ PFU/animal) respectively, except for one positive swab in a chimpanzee 6 days after vaccination [53].

Given the lack of systemic dissemination or extended excretion after immunization, contact-transfer of V-RG between vaccinated and unvaccinated animals has generally been uncommon in laboratory studies, but has been observed when contact is intimate and proximate to vaccine ingestion [54–56]. In one study, a control female fox was bitten by a male that had been vaccinated orally minutes prior. The bitten control female seroconverted and subsequently resisted rabies virus challenge [54]. In another case, two adult raccoons housed with orally immunized cage mates developed low levels of RVNA and also survived rabies virus challenge [52]. Finally, half of non-vaccinated raccoons in contact with raccoons kits orally vaccinated with V-RG (10^8.2^ PFU/animal) developed detectable RVNA, suggesting the possibility of limited horizontal vaccine transmission among kits through suckling or playing immediately after oral vaccination [57].

### Risk of recombination with orthopoxviruses present in wildlife

The potential for recombination between V-RG and other orthopoxviruses found in wildlife was initially a concern for environmental release [58]. In Europe, cowpox virus is endemic in wildlife, particularly wild rodent populations which maintain virus circulation and transmit the virus to other species, such as cats, livestock and humans [59]. A serological survey of foxes, rodents, and several other potential rabies reservoirs was conducted in Belgium. Antibodies to orthopoxviruses were detected in only two rodent species (64% of bank voles and 7% of wood mice), suggesting that cowpox virus infection is likely rare in ORV target species in Europe [60]. Experimental exposure of foxes to cowpox virus by both the oral and intradermal routes demonstrated low susceptibility to infection, suggesting a low risk of co-infection and recombination with V-RG [61]. However, a number of other orthopoxviruses have been detected in rodents and ORV target species including foxes, raccoons, and skunks (as summarized in [62]).

On-going surveillance by ORV program managers has not revealed adverse events or lesions suggestive of recombination of V-RG with laboratory or wild type poxviruses to date. However, pre-existing antibodies from raccoon poxvirus exposure has been speculated to potentially reduce antibody responses to V-RG [62]. To what extent this occurs in the field setting or what limitations this immunological interference from natural orthopoxvirus infections may place on ORV program effectiveness is not known.

### Safety in target species

The safety of V-RG has been demonstrated in a wide variety of target and non-target species in both laboratory and field studies. V-RG administration to red foxes (*V. vulpes*) by multiple routes (including oral, intradermal, gastric, and subcutaneous) was not associated with adverse events across a wide dosage range (up to 10^9.1^ TCID_50_/animal), for periods up to 18 months [54, 63, 64]. Repeated administration of V-RG FMP baits (1 bait/day for 3 days at a dose of 10^8^ TCID_50_ V-RG/bait) was well tolerated by young red foxes [65]. V-RG was similarly tested for safety in adult arctic foxes (*Vulpes lagopus*), gray foxes, coyotes, raccoons, skunks, jackals, and raccoon dogs without observation of adverse events (Table 1). Potential for enhanced neurotropism of the vaccine or allergic encephalitis secondary to vaccine administration were specifically assessed in raccoons vaccinated orally with V-RG. Neither abnormal clinical behaviour nor cytologic abnormalities in the cerebrospinal fluid were observed in these animals [66].

**Table 1:** **Safety testing of V-RG in current or potential primary target species administered by various routes**

Species	No. of animals	Route^a^	Dose per animal^b^	Observation period (days)	References
Red fox	2	i.d.	10^8.0^ PFU	28	[54]
(*Vulpes vulpes*)	2	s.c.	10^8.0^ PFU	28	[54]
	4	p.o. scarified	10^8.0^ PFU	28	[54]
	4	p.o.	10^4.0^ PFU	28	[54]
	4	p.o.	10^6.0^ PFU	28	[54]
	4	p.o.	10^8.0^ PFU	28	[54]
	5	p.o. in bait	10^8.0^ PFU/bait	28	[54]
	8	p.o. in bait	10^9.1^ TCID_50_/bait	90	[63]
	6	intragastric	10^9.1^ TCID_50_	90	[63]
	2	i.d. scarified	10^7.9^ TCID_50_	90	[63]
	4	p.o.	10^6^ PFU	30	[64]
	4	p.o.	10^7^ PFU	30	[64]
	4	p.o.	10^8^ PFU	30	[64]
	4	p.o. (lyophilised)	10^8.0^ PFU	30	[64]
	5	p.o. in bait	10^8.0^ PFU	30	[64]
	10	p.o. in bait	10^8.34^ TCID_50_/bait	182	[91]
	10	p.o. in bait	10^8.4^ TCID_50_/bait	182	[91]
	8	p.o in bait	10^7.6 to 9.5^ TCID_50_/bait	121	[144]
Red fox kits	13	p.o.	10^7.2^ PFU	33–360	[55]
(*V. vulpes*; silver var.)	65	p.o.	10^8.0 to 8.4^ TCID_50_	60–120	[81]
Gray fox	4	p.o.	10^6.8^ TCID_50_	90	Unpublished data^c^
(*Urocyon cinereoargenteus*)	6	p.o.	10^8.1^ TCID_50_	90	Unpublished data^c^
	6	p.o.	10^9.3^ TCID_50_	90	Unpublished data^c^
Arctic fox (*Vulpes lagopus*)	8	p.o.	10^8.0^ PFU	112	[82]
Raccoon	3	i.d.	10^7.0^ PFU	28	[52]
(*Procyon lotor*)	3	i.m.	10^7.8^ PFU	28	[52]
	1	p.o.	10^6.0^ PFU	28	[52]
	6	p.o.	10^8.0^ PFU	28	[52]
	8	p.o. in bait	10^8.0^ PFU/bait	28	[52]
	6	p.o.	10^8.0^ PFU	~180	[68]
	7	p.o. in bait	10^8.0^ PFU/bait	~180	[68]
Raccoon kits (*P. lotor*)	18	p.o.	10^8.2^ PFU	245 to 266	[59]
Coyote	4	p.o.	10^7.4^ TCID_50_	49	Unpublished data^d^
(*Canis latrans*)	8	p.o.	10^8.3^ TCID_50_	49	Unpublished data^d^
	7	p.o. in 1 bait/day for 3 days	10^8.5^ TCID_50_/bait	49	Unpublished data^d^
	10	p.o.	10^8.3^ TCID_50_	90	[68]
Skunk	8	p.o. in bait	10^9.0^ PFU/bait	90	[88]
(*Mephitis mephitis*)	8	Intraduodenal	10^9.0^ PFU	90	[88]
	4	i.m.	10^8.3^ PFU	90	[88]
	6	i.d.	10^8.3^ PFU	90	[88]
	6	p.o.	10^7.7^ TCID_50_	116	[89]
	5	p.o. in bait	10^7.7^ TCID_50_/bait	116	[89]
	5	p.o. in 3 baits	10^7.7^ TCID_50_/bait	116	[89]
Raccoon dog	10	p.o. in bait	10^8.8^ TCID_50_/bait	124	[91]
(*Nyctereutes procyonoides*)	10	p.o. in bait	10^9.0^ TCID_50_/bait	124	[91]
Golden Jackal (*Canis aureus*)	9	p.o. in bait	10^8.8^ TCID_50_/bait	160	[92]
	8	p.o. in bait	10^7.6 to 9.5^TCID_50_/bait	121	[144]
	3	p.o.	10^7.6 to 9.5^TCID_50_/bait	207	[144]
Small Asian mongoose (*Herpestes javanicus*)	5	p.o.	10^8.0^ TCID_50_	28	[93]
European badger (*Meles meles*)	6	p.o.	10^8.3^ TCID_50_	45	[56]
Vampire bat	56	p.o.	10^8.0^ TCID_50_	5 to 120	[108]
(*Desmodus rotundus*)	8	i.m.	10^7.4^ TCID_50_	31	[99]
	8	i.d. scarified	10^7.0^ TCID_50_	31	[99]
	8	p.o.	10^8.0^ TCID_50_	31	[99]
	8	Aerosol (nebulization)	≤ 10^7.4^ TCID_50_	31	[99]

^a^i.d., intradermal; i.m., intramuscular; p.o., per os (oral); s.c., subcutaneous.

^b^TCID_50_, median tissue culture infectious doses; PFU, plaque forming units.

^c^Unpublished data; USA registration dossier, VRG 95/036B.

^d^Unpublished data; USA registration dossier, VRG 94/069.

V-RG was also tested for safety in young and pregnant animals of many target species. V-RG was administered by the oral route to red fox kits and pregnant vixens without observation of poxviral lesions or illness [55, 64]. Safety was similarly demonstrated in pregnant raccoons and suckling kits, with no observation of adverse events [52]. Two pregnant raccoons were administered intra-muscular doses of V-RG within 30 days of parturition and subsequently delivered healthy litters. Vaccine was not isolated from the kits delivered after vaccine administration to females, which had rabies RVNA at birth, suggesting a passive transfer of maternal antibodies [52]. In another experiment, three 3 to 4 week old suckling raccoons were placed with their mother immediately after she received V-RG orally. All animals remained healthy, seroconverted within 28 days, and resisted rabies virus challenge [52]. V-RG has also been administered orally to 3 to 7 weeks old raccoon kits without adverse events [57].

Finally, concerns about the potential for V-RG vaccination of rabies virus-infected animals to induce a rabies virus ‘carrier state’ was examined in red foxes [67]. Vaccination of red foxes with V-RG during rabies virus incubation did alter the duration of incubation, inducing either early death compared to control animals when vaccination occurred proximate to experimental infection (i.e., vaccination 0 or 3 days after rabies virus challenge) or delayed death compared to controls when vaccination occurred later in incubation (i.e., vaccination 14 days after challenge). Thus, these data support the safety and use of RABORAL V-RG in rabies infected foxes [67].

### Safety in non-target species

In a field setting, any oral vaccine bait may be attractive to both target and non-target species. Thus, RABORAL V-RG was also broadly evaluated for safety in over 50 warm-blooded vertebrates, primarily by direct instillation of vaccine into the oral cavity but also by the intramuscular, intradermal, subcutaneous, intestinal, ocular and intranasal routes for some species to mimic potential accidental routes of inoculation in the field (see Additional file 1). Species tested include ecological competitors of raccoons and foxes (e.g., opossums, skunks, members of the *Canidae* family, and rodents) and species in contact with humans (companion animals—dogs and cats, domestic livestock—cattle and sheep, and commonly harvested game species—ducks and white-tailed deer). Safety was also assessed in scavengers and birds of prey, such as crows and members of Falconiformes and Strigiformes that might be exposed through ecological food webs [68]. Healthy adult animals, juveniles and pregnant or lactating animals were considered for some species due to the potential for increased susceptibility to adverse events in these demographic groups. No vaccine-associated morbidity or mortality was observed following V-RG exposure in animals evaluated from 20 taxonomic families (see Additional file 1).

Immunocompromised animal models were also evaluated due to the potential for increased susceptibility to adverse events for individuals with reduced immune competence. V-RG administered via the oral route did not cause disease in immunologically deficient mice; however, parenteral (intradermal, intramuscular or intraperitoneal) administration resulted in systemic and progressive vaccinia infection, although less severe than that seen for the parental vaccinia virus strain [27, 69]. Cats infected with immunosuppressive viruses like feline leukaemia virus and feline immunodeficiency virus had no detectable adverse effects regardless of administration route [27].

These experimental observations in select target and non-target species, conducted independently by European and North American teams, were corroborated to some extent by field trials in Europe [36, 44] and the USA [37, 38] with no reports of adverse events in target or non-target non-human animal species observed as part of post-baiting monitoring.

While the V-RG vaccine construct is not an attenuated rabies virus and, thus, cannot cause rabies by reversion to virulence, human exposure to the vaccinia vaccine vector may pose risks for clinical manifestations in persons having a contraindication for smallpox vaccination, including pregnant women, people with an acute, chronic or exfoliative skin condition, or people who are immunocompromised as described on the product insert [70]. For this reason, baits are labelled with a message “Rabies Vaccine Live Vaccinia Vector Do Not Disturb” applied in black ink directly on the FMP bait block or coated sachet plastic. The label also provides a toll-free telephone number for people to call if they have questions, concerns, or to report finding baits.

While intentional human ingestion of baits is likely uncommon due to the repugnant smell of the fishmeal bait material, people may have incidental skin or mucous membrane exposure to the vaccine through interactions with domestic pets attempting to consume a bait or when handling a bait which has ruptured. To assess potential health risks to humans, squirrel monkeys and chimpanzees were given 10^7.2^ to 10^9^ PFU doses of V-RG by the oral, transdermal and mucosal routes to mimic potential human exposure [53]. Poxvirus lesions were not observed in these animals at the site of exposure or systemically [53].

Lack of vaccine-associated lesions in healthy non-human primates does not rule out the potential for adverse events from V-RG exposure in immunocompromised persons. For this reason, bait distribution strategies are designed to minimize public contacts with baits. Reports of human contact with baits are relatively rare and typically involve efforts to take a bait from the mouth of a dog. For example, in France 96 human contacts with ORV baits were reported during 1992 to 1996, a period during which 8.4 million RABORAL V-RG and SAG2 (an attenuated rabies vaccine-bait product) baits had been distributed. Forty-four percent of these contacts occurred when dog owners tried to remove a bait from a dog’s mouth [71]. In the USA and Canada, reports of human contact with intact or ruptured baits have been similarly rare compared to the number of baits distributed, with few adverse reactions or illnesses associated with these reports [31, 72–74]. For example, during 2001 to 2009 in the USA 44 million RABORAL V-RG baits were distributed and exposure surveillance in 18 states recorded 296 human contacts with ruptured baits and 550 pet contacts with baits [73]. Six human adverse events were reported to the Centers for disease control and prevention during 2001 to 2009, five skin rashes or dermatological reactions of undetermined origin at the site of virus contact and one diagnosis of clinical vaccinia virus infection (1 of the 2 cases of vaccine-associated vaccinia infection described below, the first of which occurred prior to 2001) [73].

In the USA, two human exposures to RABORAL V-RG resulted in vaccinia-like infections. In both cases, exposure involved inoculation of the vaccine into fresh skin wounds while handling a dog that had recently eaten a bait [75, 76]. The first case was reported in Ohio during September 2000 in a pregnant woman aged 28 years with epidermolytic hyperkeratosis who was bitten while pulling a ruptured bait from her dog’s mouth without washing of the wound afterwards. The woman developed swelling and erythema of the arm, left axillary adenopathy, pustules and necrotic scabs at the site of the dog bite; her skin at the site of virus inoculation ultimately healed and the pregnancy followed a normal progression [75]. The second case occurred in Pennsylvania during August 2009 in a 35-year-old woman receiving immunosuppressive medications for inflammatory bowel disease. The woman was exposed to the vaccine through a patch of abraded skin after handling a ruptured bait without washing her hand after exposure. She developed localized cutaneous lesions at the site of vaccine contact. She was treated with human vaccinia immune globulin intravenously and an experimental antiviral agent and recovered [76].

## Immunogenicity and efficacy in controlled laboratory trials

### Foxes

The red fox (*V. vulpes*) is a primary reservoir species in Europe and Canada [77] while the gray fox (*Urocyon cinereoargenteus*) is the most common fox reservoir species in the USA except in parts of Alaska [78, 79]. Rabies virus is highly pathogenic in red foxes [77]. V-RG is efficacious at inducing immunity in red foxes by intradermal, subcutaneous, and oral routes [63]. Adult foxes were fully protected against rabies virus challenge performed 12 and 18 months after oral administration of V-RG [64]. V-RG is also effective at immunizing young animals. The majority of red fox kits (6 to 12 weeks old at time of vaccination) seroconverted and resisted rabies virus challenge undertaken up to 12 months after oral administration of V-RG [55, 65]. The minimum duration of immunity measured for V-RG in the red fox (12 months in kits and 18 months in adults) corresponds well to the typical fox lifespan in the wild (1 to 3 years) [64].

In the field many fox kits in ORV distribution areas may be offspring of immunised vixens producing a potential situation for interference from maternally-derived antibodies in these kits in subsequent vaccination campaigns. Investigation of maternal antibodies interference in captive fox kits demonstrated that RVNA can be transferred to kits of vaccinated vixens, but RVNA levels declined 45 to 75 days after birth, suggesting that maternal antibody interference with immunization of kits should be limited to 4 to 6 weeks after birth. Kits born to vaccinated vixens orally vaccinated at 30 days of age had comparable RVNA production as kits of unvaccinated vixens vaccinated on the same schedule and all vaccinated kits survived rabies virus challenge at 5 months of age [80]. Similar RVNA titres and protection from rabies virus challenge were also observed in kits vaccinated at 90 days of age regardless of maternal vaccination status prior to birth [81].

An anamnestic response was observed in foxes after an oral booster vaccination at 35 days after a first vaccination with V-RG. However, this response was of limited duration, suggesting that a second dose of RABORAL V-RG is not required to achieve sufficient individual-level immunity to rabies virus when orally vaccinated with this vaccine [80]. Orally vaccinated foxes have survived rabies virus challenge despite not having detectable RVNA, suggesting a possible role for cell-mediated protective immunity [54, 64].

A close relative of the red fox, the arctic fox (*V. lagopus*), is considered the primary rabies virus reservoir in northern and western Alaska, in the high Canadian arctic and other regions within this species’ natural range [79]. V-RG elicits a strong RVNA response in this species sufficient for protection against rabies virus challenge [82].

The gray fox is a more distant relative of the red fox and is an important rabies virus reservoir in portions of the southern and southwestern USA [78, 83]. V-RG is immunogenic in gray foxes by the oral route with development of RVNA titres comparable to those seen in vaccinated red foxes and resulting in survival of a rabies virus challenge (unpublished data; USA registration dossier, VRG 95/036B). Due to gray foxes’ poor adaptability to captivity, a caged gray fox efficacy trial and rabies virus challenge, as required to license veterinary biologicals in the USA, has not been performed as of this writing. However, long-term (20 years) experimental use has allowed for distribution of RABORAL V-RG in west-central Texas, USA. This program has demonstrated the field effectiveness of RABORAL V-RG for controlling rabies virus circulation in gray foxes [83] and the gray fox rabies virus variant has nearly been eliminated from Texas as of this writing [83, 84]. Cumulative biomarker, vaccine safety and serological data from decades of field use combined with rabies case reporting since 1996 have documented the use of this vaccine to essentially eliminate the gray fox rabies variant from Texas. The last spill-over case of rabies related to the gray fox variant as of this report was in a cow in 2013 [84] (Additional file 2).

### Raccoons

Since 1990, the raccoon (*P. lotor*) has been the primary rabies reservoir in the eastern USA. Raccoons are the most frequently reported rabid wildlife species in the USA, accounting for 32.4% (includes all rabies variants) of rabies cases in 2014 [78]. Raccoon rabies constitutes a significant public health concern in North America due to its impact on large metropolitan areas, as well as the close relationship between raccoons and humans in suburban environments. Moreover, spill-over of the raccoon rabies virus variant to other non-reservoir species occurs frequently. The raccoon rabies virus variant (*n* = 1822) accounted for 30.2% of all animal rabies cases in the USA reported during 2014, with skunk (*n* = 1588) and bat (*n* = 1756) variants making up a large portion of the remainder (26.3 and 29.1%, respectively) [78].

V-RG is immunogenic for raccoons by the intradermal, intramuscular and oral routes [52]. A single oral dose of V-RG delivered in an experimental sponge bait format protected raccoons against rabies virus challenge, with 100% survival at 28 days post-vaccination and 80% survival at 205 days [16]. Efficacy of the vaccine was apparently not enhanced by buccal scarification or by the administration of booster doses [52]. Interestingly, some raccoons, as was observed in foxes, immunized with V-RG survived rabies virus challenge despite a low RVNA titre at the time of rabies virus inoculation, whereas others with high titres succumbed to a virulent challenge dose [52], a phenomenon also reported after oral immunization of raccoons with the SAD-B19 attenuated rabies virus vaccine [15].

Efficacy of V-RG in free-ranging raccoons was evaluated during the first RABORAL V-RG field trials in the USA (1990) on Parramore Island, Virginia. Free-ranging raccoons were captured and challenged with rabies virus 7 months after RABORAL V-RG was distributed at a very high density on the island [38]. Control raccoons were trapped on Revel’s Island, close to Parramore Island. All but one of the Parramore Island raccoons were positive for tetracycline marking, indicative of bait consumption, and 7/18 had RVNA > 0.5 IU/mL (range 0.6 to 54.0 IU/mL) on the day of rabies virus challenge. Ten of 11 (91%) of control raccoons from Revel’s Island succumbed to rabies virus challenge, whereas 14 of 18 (77.8%) Parramore Island raccoons survived. An anamnestic response was observed in all 14 surviving Parramore raccoons as a result of the rabies virus challenge, but not in the raccoons which succumbed to rabies [85]. This initial trial demonstrated that the bait-vaccine could effectively immunize raccoons in the field setting, however oral vaccination effectiveness in subsequent field studies has been quite variable and depends on both programmatic and ecological factors (see additional discussion of field effectiveness in Sect. 5). Similar to foxes, V-RG is also immunogenic in very young raccoon kits [57] (Additional file 2).

### Coyotes

Rabies emerged as a significant problem in coyotes (*C. latrans*) in South Texas, USA during the late 1980s, likely as a result of spill-over from infected dogs [32]. An ORV program was initiated during 1995 to stop the spread of a canine variant epizootic in South Texas before infected coyotes entered major human population centres in the state [83]. Annual application of approximately 1 million doses of RABORAL V-RG during January of each year (1995 to 2005) resulted in the rapid decline and elimination of the canine rabies virus variant from Texas coyote populations. Today, a maintenance zone continues to immunize coyote populations along the Mexico-USA border. Prior to use in the field, oral vaccination of captive coyotes with V-RG demonstrated safety, immunogenicity, and efficacy in this species (work performed under a collaborative agreement between the Texas Department of State Health Services, Austin, TX; The Centers for Disease Control and Prevention in Atlanta, GA, USA and Rhone Merieux, Inc., Athens, GA, USA; unpublished data; USA registration dossier, VRG 94/069) (Additional file 2).

### Skunks

The striped skunk (*M. mephitis*) is also an important rabies reservoir in North America, particularly in the Central Plains of the USA and Canada. During 2014, skunks were the second most common wild carnivore species reported rabid in the USA [78]. Spill-over of raccoon rabies virus variant commonly occurs in striped skunks and this species may play a role in maintaining circulation of the raccoon rabies virus variant [86, 87]. Skunk populations maintain circulation of different skunk rabies virus variants in the central and southwestern regions of the USA, as well as in California [78]. Compared to other rabies hosts, skunks have different foraging behaviours, are relatively resistant to oral immunization by attenuated rabies virus vaccines and have been documented to develop clinical rabies when administered these modified-live viruses [10, 17].

V-RG is effective at immunizing and protecting striped skunks from rabies virus challenge when administered by different delivery routes (oral sponge baits, scarification, intramuscular injection and intraduodenal injection) [88, 89]. Six of 7 skunks that consumed an experimental V-RG-laden sponge seroconverted (RVNA range of 0.17 to 4.61 IU/mL on day 28 post-vaccination) and 5 of 7 skunks resisted a rabies virus challenge performed 90 days post-vaccination [88]. However, skunks appear to have difficulty ingesting vaccine from the plastic sachet used in current RABORAL V-RG product formats—while 67% (4/6) of skunks receiving a complete (1.5 mL) dose by oral instillation were protected from challenge, only 20% (1/6) survived challenge when provided the vaccine within a coated sachet bait for voluntary consumption [89]. Skunks offered V-RG in coated sachets while housed in an elevated cage setting with mesh floors did not develop detectable RVNA and immunization success was not enhanced by offering multiple baits. Poor vaccination efficiency was attributed to vaccine spillage when skunks manipulated the bait during feeding [89]. While V-RG is immunogenic in striped skunks, a different method of delivery (possibly modified baits, different bait distribution strategies, or both) may be the critical link for achieving effective oral vaccination of this species in the field (Additional file 2).

### Other species

#### Raccoon dogs

The racoon dog (*N. procyonoides*) is an Asian species introduced by the fur industry into western Russia around 1920. Raccoon dogs have recently emerged as a secondary rabies host after the red fox in several European countries and are now thought to play a major role in the epidemiology and epizootiology of the disease in eastern and northern Europe [90].

Caged raccoon dogs (*n* = 20) offered RABORAL V-RG in the USA manufactured-FMP bait format developed high RVNA titres and survived a rabies virus challenge performed 124 days after vaccination, whereas all rabies virus challenge controls (9/9) died of rabies [91] (Additional file 2).

#### Jackals

The golden jackal (*C. aureus*) is native to north and northeast Africa, southeast and central Europe, Asia Minor, the Middle East, and Southeast Asia. During the 1950 to 1970s, golden jackals were a primary rabies reservoir in Israel [92].

Nine golden jackals administered RABORAL V-RG in an FMP bait were challenged 160 days post-vaccination with a local jackal rabies virus isolate. Seroconversion was observed in 44.4% of vaccinated jackals on day 150 post-vaccination and 77.7% of vaccinated jackals survived the rabies challenge that killed all ten controls [92] (Additional file 2).

#### Mongooses

The small Asian mongoose (*Herpetes javanicus*) was introduced throughout the Caribbean in the mid-1800s as an ill-advised attempt to control rodent populations in sugarcane fields. Mongooses are now the main reservoir of rabies in the Caribbean with numerous human exposures and are a source of spill-over to dogs and other susceptible mammals. Thirty-two rabid mongooses were reported during 2014 in Puerto Rico [78]. Five small Asian mongooses administered V-RG by direct oral instillation did not develop detectable RVNA. Four of the five vaccinated mongooses and all controls succumbed to rabies when challenged at 28 days post-vaccination [93]. Further work is needed to determine how to effectively vaccinate mongoose by the oral route against rabies [94]. This species may pose similar challenges to effective vaccine delivery as seen in skunks due to relatively small mouth size or differences in feeding ecology; both bait modifications and consideration of new bait distribution approaches may be required to effectively reach this species in the field setting (Additional file 2).

#### Badgers

European badgers (*Meles meles*) are very sensitive to the red fox variant of rabies virus and can excrete high amounts of virus in saliva [95]. While not a primary reservoir, badgers are relatively commonly infected with rabies virus through spill-over from other hosts, making them a potential target for ORV.

Badgers did not show high antibody responses or protection against challenge when given a 10^8.3^ TCID_50_ dose of V-RG by the oral route. Only 2 of 6 badgers developed RVNA titres ≥ 0.5 IU/mL and only 2 of 5 vaccinated badgers survived rabies virus challenge on day 45 post-vaccination [56]. Additional study is needed to determine if badgers may require a higher dose of V-RG than other target species or if other barriers to effective oral immunization exist in this species (Additional file 2).

#### Bats

Considerable gaps still exist in our collective knowledge regarding rabies and other viruses in bats. Bats are important lyssavirus reservoirs globally, and particularly in the Americas. During 2014, bats comprised 29.1% of reported animal rabies cases in the USA [78]. In Latin America, the common vampire bat (*Desmodus rotundus*) is a primary wild rabies virus reservoir affecting humans, livestock and other species [96].

Currently, vampire bat rabies control methods frequently include bat population reduction through the use of an anticoagulant paste applied on the back of captured bats, which upon release spreads the poison to the colony through mutual grooming. However, these methods have only achieved short-term respite in limited areas [96]. Further, recent studies suggest that local population reduction of vampire bats may actually worsen rabies risks to humans and livestock by shifting bat population demographics to higher numbers of juveniles and sub-adults, which are more likely to circulate rabies virus [97].

V-RG was found to be immunogenic when experimentally administered to vampire bats by the oral, intramuscular, intradermal and aerosol routes. High protection rates against a rabies virus challenge were obtained after oral administration of a relatively high vaccine dose [98–100]. A V-RG concentrated suspension mixed with neutral Vaseline paste was applied on the back of one bat that was housed with other unvaccinated bats thus demonstrating a potential for co-opting vampiricide distribution strategies to immunize bat colonies [100]. Of bats indirectly vaccinated with V-RG via exposure to bats carrying the vaccine paste, 81% (17/21) survived rabies virus challenge [100].

Control of rabies in vampire bats to prevent human and livestock exposure remains a public health challenge but these studies suggest that vaccination via the oral route may be feasible and could contribute to improved prevention of bat rabies in affected countries. However, regulatory approval process for environmental release of ORV targeting for bats will also need to be addressed (Additional file 2).

## Effectiveness of RABORAL V-RG in the field

### Europe

The large western European epizootic of rabies in red foxes (> 75% of reported rabies cases) experienced during the 1980s which spurred the development of ORV campaigns in Europe most likely originated from the Russian-Polish border in 1935 [77, 101]. The first large scale ORV field trial targeting red foxes occurred during 1978 in Switzerland and used the SAD attenuated strain of rabies virus in edible baits placed at fox dens [7]. This pivotal trial was soon followed by ORV use in other western European countries. Most campaigns were performed during spring to target adult foxes mainly when population density was lowest (whelping takes place during early spring) and again in autumn (September–October) to reach both adults and young foxes when they begin to disperse.

RABORAL V-RG was licensed for use in the red fox in France in 1995 and in Belgium and Luxembourg in 1996. From 1989 to 2005, approximately 10.5 million RABORAL V-RG doses were distributed targeting red foxes which contributed to the elimination of terrestrial rabies cases in these countries. Additionally, since 2006 more than 30 million doses of RABORAL V-RG have been distributed in the Ukraine. The effectiveness, utility, safety and genetic stability of RABORAL V-RG were first demonstrated in western Europe. Early fox ORV campaigns identified key program variables (e.g., line spacing and bait density) as well as addressed concerns about environmental release of a genetically modified vaccine.

#### France

Canine rabies predominated in the first half of the twentieth century in France but began to decline by the early fifties in response to effective integrated programs anchored by parenteral vaccination of dogs. France became free of rabies in carnivores during 1960, but rabies re-emerged in north-eastern France during 1968 when a rabid fox was detected in Moselle near the Franco-German border. Rabies spread through the fox population in northeast France from 1968 to 1974 at a mean rate of 30 to 40 km/year [101]. France initiated a limited ORV program in 1986 in collaboration with Belgium, Luxembourg, and Switzerland. Limited areas within the Lorraine region and in the French Alps were vaccinated with SAD-B19 vaccine baits, but despite encouraging early results, these areas were rapidly re-infected from other unvaccinated or under-vaccinated areas [101–103]. Despite the presence of potential geographic barriers, even large rivers (e.g., the Seine and the Loire) did not prevent rabies spread.

**Figure 3:**
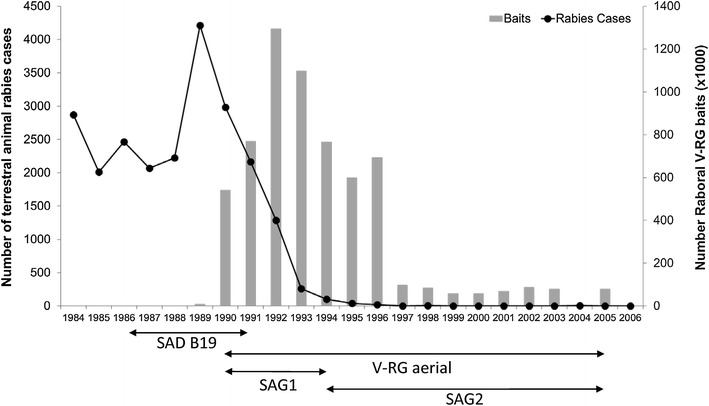
**Rabies prevalence in terrestrial animals and V-RG bait distribution volume, France—1984 to 2006.** Wildlife ORV efforts began during 1986 using an attenuated rabies virus vaccine (SAD-B19) and continued from 1990 through 2005 using a combination of RABORAL V-RG and attenuated rabies virus vaccines (SAG1 and SAG2) (Data sources: [47, 101, 107, 150]).

To address a progressing rabies outbreak front, a continuous 50 km wide ORV barrier was established in 1990 from the English Channel to the Swiss border to protect the southwest of France, which remained free of rabies. The barrier was enlarged to the north and east to cover the entire affected area from autumn 1992 to 1997 [101–103]. From 1997 to 2000, ORV campaigns were conducted along French borders with Switzerland and Germany [103]. From 2001 to 2003 and during 2005, ORV occurred over a limited area of 5300 km^2^ bordering Germany [45]. Rabid fox cases declined under this program and no rabies cases in wild carnivores have been recorded since December 1998 [101] (Figure 3). France has been recognized as free of rabies in wild carnivores since 2001 [103].

Over the course of this ORV program, attenuated rabies vaccines were first used in France—SAD-B19 from 1986 to 1991 and SAG1 then SAG2 from 1990 to 2005—and then RABORAL V-RG was used from 1989 to 2005 (Figure 3). Baits were distributed by helicopter in spring and autumn, initially at a density of 13 baits/km^2^ and then at 20 baits/km^2^ due to an increase in fox population. The helicopter was preferred to light aircraft for more accurate delivery, which was advantageous in mountainous and heavily populated zones. In addition, helicopters could be used in less favourable weather conditions [102]. A comparison of field effectiveness of the three vaccine-baits used in France between 1988 and 1994 (2 attenuated oral rabies vaccine and V-RG) suggested that RABORAL V-RG was the most efficient for summer distribution, and resulted in fox rabies elimination in a non-alpine region after only two campaigns [102]. The higher efficiency was attributed at that time to a better environmental stability of the RABORAL V-RG vaccine and baits [102].

Different strategies were evaluated in the field to increase the efficiency of ORV, especially in fox kits. In spring, kits are the largest group in a fox population, but are also the most difficult group to vaccinate according to bait uptake estimates using tetracycline biological marker. Only 33 to 65% of kits consumed RABORAL V-RG baits compared to 64 to 86% of adults during spring campaigns, and only 52 to 86% of kits compared to 76 to 85% of adults during autumn campaigns [102]. A summer vaccination campaign was conducted to reduce the period of susceptibility of fox kits and reach older fox kits, when they begin to forage by themselves away from their dens. This campaign led to a significant increase in bait uptake by fox kits, but was found to be less efficient for decreasing rabies prevalence than campaigns carried out in spring or autumn. Bait distribution at den entrances significantly increased uptake by fox kits, but proved difficult to organize and costly. Bait distribution during spring, autumn, then spring, was more efficient in foxes than distribution during autumn, spring, and then autumn [48].

A cost-effectiveness analysis comparing two strategies for wildlife rabies control in Europe where foxes are the primary reservoir showed that ORV became beneficial over population reduction after the fourth year of ORV application [104].

#### Belgium

Fox rabies entered Belgium from Germany during 1966 and expanded west and south to reach the Meuse and Sambre valleys, which appeared to constitute a natural barrier to the spread of rabies [44]. The southern infected area reached 10 700 km^2^ in size [103]. During 1986 to 1987, Belgium participated in an international field trial of ORV of foxes using the SAD-B19 vaccine, which was distributed over a 2100 km^2^ area located around the border with Luxembourg [104, 105]. Small-scale field trials conducted in southern Belgium in October 1987 (6 km^2^) [19] and September 1988 (435 km^2^) [44] demonstrated the safety of RABORAL V-RG in the field setting. The national ORV program began in 1989.

**Figure 4:**
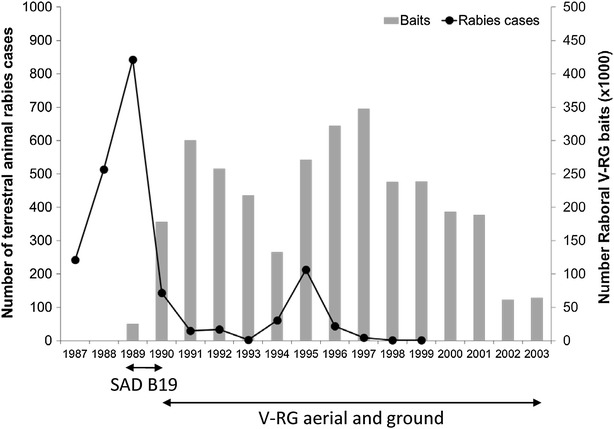
**Rabies prevalence in terrestrial animals and V-RG bait distribution volume, Belgium—1987 to 2003.** Wildlife ORV efforts began during 1989 using an attenuated rabies virus vaccine (SAD-B19) and continued from 1990 through 2005 using aerial and ground distribution of RABORAL V-RG (Data sources: [47, 101, 107, 150] and personal communication, B. Brochier).

During autumn 1989 and spring 1990, SAD-B19 and RABORAL V-RG were used, then RABORAL V-RG was used exclusively from autumn 1990 (Figure 4). Vaccine-baits were distributed by air (helicopter or airplane) at a mean density of 15 baits/km^2^ [36, 47, 106]. From 1989 to 1991, five vaccination campaigns covered the entire infected area (10 000 km^2^), leading to an initial decrease in documented rabies cases in foxes and elimination of the disease from the majority of the affected area [106]. Restricted campaigns were conducted along the French border from 1992, resulting in a further decrease in rabies cases [101].

During 1994, a reinfection occurred in areas previously freed from rabies near the French border, resulting in a change in strategy during 1996 [101, 104]. Two aerial distributions were performed during the cold season (March–April 1996 and November–December 1996), the baiting density was increased to 17 baits/km^2^ (due to an increase in fox density), the distribution was improved through the use of the global positioning system (GPS) technology and baits were also distributed at dens (10 to 20 baits/breeding den) [101, 107]. The number of rabies cases decreased rapidly from 1996 to 1999. The last fox rabies case was detected during April 1998 and in a cow in July 1999 [101, 103, 107] (Figure 4). Belgium was declared officially free of fox rabies in 2001 [108]. Until the end of 2003, two ORV campaigns were carried out per year over a limited area (1800 km^2^) along the border with Germany [103].

Bait uptake based on tetracycline detection in bones during 1990 to 2000 ranged from 51 to 95% after spring campaigns in adult foxes and from 48 to 73% in young foxes and from 54 to 83% in adults after autumn campaigns. Higher bait uptake was recorded after spring campaigns when the density of the adult fox population is the lowest and kits have not dispersed, and after the use of the GPS [107]. From 1995 to 2000, RVNA were detected after spring campaigns in 66 to 87% of adult foxes compared to 51 and 77% after autumn campaigns [107].

#### Luxembourg

**Figure 5:**
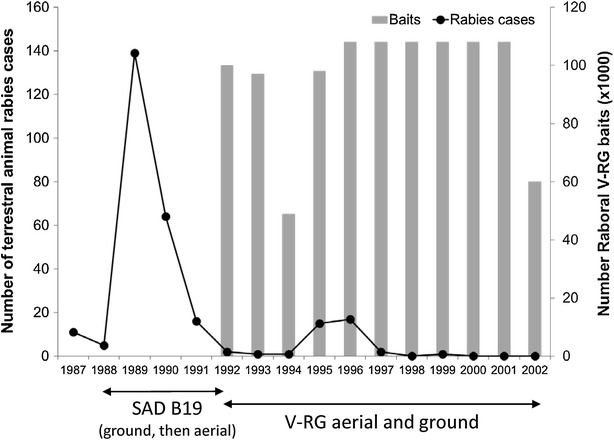
**Rabies prevalence in terrestrial animals and V-RG bait distribution volume, Luxemburg—1987 to 2002.** Wildlife ORV efforts began during 1988 using an attenuated rabies virus vaccine (SAD-B19) and continued from 1992 through 2002 using aerial and ground distribution of Raboral V-RG (Data sources: [47, 101, 107, 150] and personal communication B. Brochier).

Sylvatic rabies invaded the Grand-Duchy of Luxembourg in 1966 and established throughout the country (2586 km^2^), despite control efforts by various means (e.g., fox den gassing and culling and compulsory vaccination of dogs). An international field trial conducted during 1986 and 1987 using SAD-B19 baits (consisting of 3 campaigns) by Belgium, France and Germany created an 18 000 km^2^ immune zone around Luxembourg. Baits were distributed manually (15 baits/km^2^) [101]. From 1988 to 2001, extensive biannual vaccination campaigns were carried out, except in May 1988 (200 km^2^), May 1989 (400 km^2^) and in 1994 (1 campaign) [101]. RABORAL V-RG was used instead of SAD-B19 beginning in 1992 at a density of 18 to 20 baits/km^2^ [47]. In 1990, manual distribution was replaced by helicopter [101]. Distribution at dens was performed at the beginning of June by hunters [103]. The last rabies case was detected in a pony in January 1999 in the north of the country [101] (Figure 5). As for Belgium, Luxembourg was declared officially free of rabies in 2001 [108]. The last vaccination campaign occurred in 2002 [103].

#### Ukraine

Public health infrastructure in Ukraine combats co-existing urban and sylvatic rabies cycles under difficult political and social conditions. Roaming dogs and high reservoir wildlife densities (5 to 6 foxes/100 km^2^) contribute to repeated focal epizootics. Spill-over of rabies occurred with more than half (56.8%) of 2015 cases reported in domestic animals [109]. Initial use of RABORAL V-RG in Ukraine used doses imported from the USA. From 2006 to 2008 ORV programs targeting foxes occurred in 16 regions using approximately 27 million doses (9 million per year at 15 baits/km^2^). After 2009 the program size was reduced due to economic reasons [109]. Although rabies is not controlled nationally, sustained efforts (2005 to 2015) have shown substantial progress in four border regions with Russia (Luganska, Donetcka, Poltavsak, Sumska) covering 85 659 km^2^ using baiting densities of 15 to 20 baits/km^2^. Wildlife and domestic rabies case reports from these regions have declined from a peak in 2007 (367 wildlife/627 domestic) to 28 reported wildlife cases and 154 domestic cases in 2015 (personal communication by V. Solotchuk, Ukrvetprompostach, Ltd., Ukraine). An ORV program at Ukraine’s western border with Poland covering approximately 26 400 km^2^ is operated in concert with European Commission rabies eradication efforts [110].

### North America

Wildlife rabies prevention programs were implemented in North America as rabies outbreaks emerged near population centres in eastern Canada and the Atlantic coast of the USA. The first Canadian oral rabies vaccine program began during 1989 targeting foxes with attenuated ERA rabies virus-filled baits [24]. In parallel, the USA began evaluating the potential use of a vaccinia-vectored recombinant vaccine bait (a precursor to the current commercial product) in raccoons during 1990 in response to a raccoon rabies outbreak which emerged during the mid-1970s following suspected translocation of infected raccoons from an enzootic region (Florida, Georgia, eastern Alabama, and southern South Carolina, USA) to western Virginia and southern West Virginia, USA [16, 111–113].

Raccoon rabies virus variant continued to spread through New England and into upstate New York with the first reported rabid raccoon in Canada detected in Ontario during 2009. North American scientists and government agencies decided to address rabies outbreaks along international borders [i.e., Canada (raccoon variant) and Mexico (canine variant)] thus leading to the formation of the North American Rabies Management Plan. The plan was signed during October 2008 by representatives of the USA, Canada, Mexico and the Navajo Nation pledging to address wildlife rabies across international boundaries and disciplines [114]. As of this writing, wildlife rabies control programs using ORV continue annually in Canada and the USA, where meso-carnivores and bats are the primary reservoirs of public health concern, while Mexico is focused on control and elimination of canine rabies through dog vaccination campaigns and monitoring and controlling the emerging threat of vampire bat rabies.

#### United States of America

Multiple wildlife species (e.g., raccoons, skunks, foxes and bats) are potential reservoirs of rabies for both humans and domestic animals in the USA [78]. Terrestrial species-associated rabies virus variants occur in distinct geographic areas: raccoon rabies virus variant in the eastern USA, skunk rabies virus variants in the central USA and California, fox rabies virus variants in Texas, Arizona, New Mexico and Alaska, and dog-mongoose rabies virus variants in Puerto Rico [78].

The first USA-based V-RG field trial occurred during August 1990. Prototype cylindrical fishmeal baits containing V-RG and tetracycline biomarker were distributed by hand at a high density (1000 baits/km^2^) on a barrier island (Parramore Island, Virginia) to determine if an isolated population of free-ranging raccoons could be effectively vaccinated by the oral route and to monitor for potential adverse vaccine effects in target and non-target species [38]. Thirty days later evidence of tetracycline biomarker was detected in bone samples of 47/56 (84%) raccoons in the vaccination area [85]. In addition, RVNA were detected in 57% of raccoons [38]. No adverse effects or orthopoxvirus-like lesions were observed in raccoons or other observed non-target species [38]. Fourteen of 18 raccoons collected from the island survived a rabies virus challenge 7 months after consuming the experimental baits. All surviving raccoons and three of the four not surviving challenge were biomarker positive [38].

Subsequently, a second safety study was conducted on State Gamelands #13, in Pennsylvania during 1991 [115] and a third safety and initial field effectiveness trial followed in Cape May, New Jersey during 1992 to 1994 against an advancing raccoon rabies epizootic front [49].

Over the next 10 years, ORV campaigns targeting raccoons were implemented by a number of state or county agencies in Massachusetts, Florida, New York, Vermont, Ohio, New Jersey, and Maryland [40, 49, 113, 116–118]. RABORAL V-RG was approved for use in raccoons in 1997 as a United States Department of Agriculture (USDA) licensed veterinary vaccine. A federal cooperative ORV program began in 1998 led by USDA-Wildlife Services to coordinate ORV efforts already underway in Ohio and Vermont, and participate as a primary co-operator in the state-led Texas ORV programs to ensure harmonization with national rabies management objectives (i.e., preventing wildlife rabies from spreading into naïve areas of the USA). Northern New York was added to the federal program in 1999, with Cornell University leading initial coordination efforts. The raccoon ORV program’s goal of preventing the variant from spreading westward supported expansion into to Pennsylvania; West Virginia; eastern Tennessee, Alabama and Georgia; and North Carolina to prevent the endemic rabies variant from spreading westward beyond the Appalachian Mountains. ORV zones were expanded into New England to mitigate risks of raccoon rabies spreading north to Canada.

The federal program integrated natural terrain features (e.g., rivers, lakes, and poor raccoon habitat along mountain ridges) as anchor points for ORV zones from the Ohio shore of Lake Erie south into central Alabama below Birmingham. Vaccine-filled baits were distributed once a year (August–September) in campaigns at a target density of 75 baits/km^2^ using airplanes, helicopters and hand placement of baits to create vaccination zones of at least 40 to 50 km in width [119]. In addition, contingency response actions have been used to maintain the integrity of established ORV zones against new outbreaks, such as in 2004 in northeast Ohio between the established ORV zone and the eastern suburbs of Cleveland [120, 121]. Contingency actions consisted of added features (e.g., more than one ORV baiting/year, higher density baiting or trap-vaccinate-release (TVR) of raccoons using inactivated rabies vaccine, or combinations of these methods) to bolster the effectiveness of ORV zones [119, 122, 123].

Decades of field experience have proven that many variables affect the field effectiveness of an ORV programs for any particular species. However, optimizing and evaluating raccoon ORV programs has proven particularly challenging due largely to the diversity of habitats where raccoons and rabies management occurs as, well as the complexity and the adaptability of this species to thrive at varying population densities across large geographic areas affected by raccoon rabies. The expanse and heterogeneity of raccoon habitats and other factors (e.g., presence of skunks in raccoon variant endemic areas) contribute to the challenge of achieving the USA strategic goal of stopping the spread and eventually eliminating raccoon rabies at the local, regional and national level [122]. Currently, ORV campaigns in the USA typically distribute baits once annually in the fall, typically at a target density of 75 baits/km^2^ in rural areas and at 150 baits/km^2^ in urban and more developed areas.

For these large-scale programs in the USA, RVNA serology has been an important tool for evaluating success of ORV campaigns for reaching raccoon populations. During the 2008 to 2011 period, using blood samples collected 4 to 12 weeks post-baiting, the proportion of raccoons reported with RVNA ≥ 0.05 IU/mL ranged annually from 29% ± 14% to 37% ± 17%, with wide variation in ranges [122, 123]. Median raccoon age was 1 year, underscoring the likely importance of baiting in late summer or early fall to target juvenile raccoons that may disperse, as the mortality from rabies in this cohort may be relatively high [122].

Antibody levels peak in raccoons at 4 to 6 weeks after oral rabies vaccination and then decline [38, 123]. Thus, observed seroprevalence rates from samples collected after 6 weeks post-baiting may incompletely reflect existing population-level immunity [123]. Despite declines in detectable RVNA, raccoons that have been exposed to the vaccine may remain protected against rabies virus infection for months after vaccination [52]. Conversely, high RVNA seroprevalence in raccoon populations post-baiting may not extinguish rabies virus circulation sufficiently due to other demographic and ecological factors. For example, skunk populations are thought to contribute to rabies virus circulation in many raccoon rabies affected areas [86, 87].

Thus, interpretation of serologic surveys in raccoon populations post-baiting is difficult and may reflect a variety of programmatic (e.g., number of annual bait distributions, bait density, flight line spacing); demographic (e.g., raccoon density, rate of population turnover, migration rate) and ecological factors (e.g., urban versus rural habitat, presence of skunks, availability of competing foods and the presence of bait competitors). Baiting strategies to improve ORV bait uptake in raccoons have included varying bait densities [111], use of bait stations [124], and pulse or cluster baiting to potentially increase bait update by juveniles foraging in family groups [122].

RABORAL V-RG has been instrumental for control of coyote and gray fox rabies in Texas. During 1988 to 1994, 531 cases of canine variant rabies were reported in Texas (270 in coyotes and 216 in domestic dogs). The epizootic began in 1988 in South Texas, along the USA-Mexico border in unvaccinated dogs then coyotes, and expanded to include 18 contiguous counties [125]. The emergence of the canine variant in coyotes in South Texas and two associated human deaths prompted Texas to enhance state rabies control measures [125]. In 1995 an ORV program distributed RABORAL V-RG in an arc-shaped band over a 24-county area approximately 200 km north of Laredo, then as case numbers declined, baits were distributed annually progressively moving the vaccination zone southward toward the Rio Grande River, thereby decreasing the size of the rabies-infected area [83, 119, 125]. Baits targeting coyotes were distributed aerially along GPS transect lines at a density of 27 baits/km^2^. Subsequent annual ORV campaigns were conducted in winter (January) due to extreme heat in south and west-central Texas and the potential competition for baits from fire ants (*Solenopsis invicta*) during the summer. Decreased availability of food in the winter may increase consumption of baits by coyotes [125, 126].

**Figure 6:**
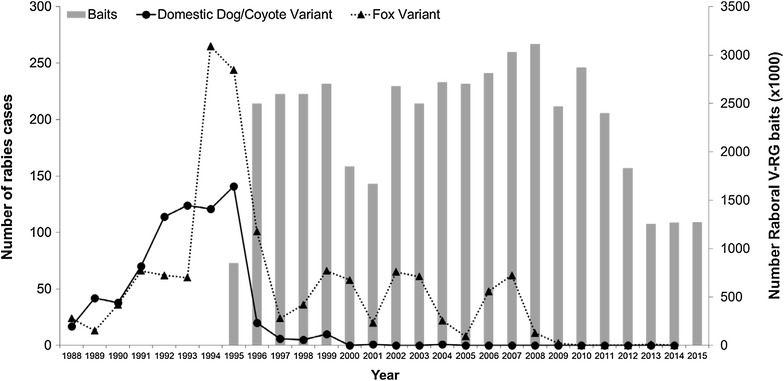
**Cases of domestic dog/coyote and gray fox rabies virus variants and V-RG bait distribution volume, Texas—1988 to 2014.** Wildlife ORV efforts in Texas began in 1995 for coyote and 1996 for gray fox, both programs using RABORAL V-RG (Data source: modified from [128] with raw data provided by the Texas Department of State Health Services for production of graphics).

Between 1995 and 2003, 70% of coyotes sampled in South Texas were tetracycline-positive and 56% had detectable RVNA [83]. After 2003, the coated sachet format replaced the fishmeal bait and serology alone was used for post-baiting monitoring. The number of rabies cases fell from 122 in 1994 (before ORV began) to 10 cases in 1999, illustrating the ORV program’s effectiveness in coyotes (Figure 6). Two rabid dogs were detected (1 in 2001 and another in 2004) near the USA border in Mexico; however, no domestic dog/coyote rabies virus variant cases have been detected since 2000 in South Texas [126] (Figure 6). Today, the canine rabies is considered eliminated from the USA [111, 127]. A barrier ORV zone 30 to 65 km wide using RABORAL V-RG is maintained along the Texas-Mexico border to reduce the risk of re-entry of canine rabies virus variant into the USA [119].

RABORAL V-RG has also been experimentally applied to control-rabies in gray fox populations in Texas. From 1988 to 1995, 764 cases caused by a gray fox rabies virus variant were reported from Texas (411 in gray foxes) [83]. The epizootic began in west-central Texas and expanded to 46 contiguous counties in the west-central part of the state [83]. During 1996, an ORV program was initiated by encircling the epizootic area with a 32 km-wide ORV zone with an added 16 to 24 km vaccination buffer along the northern and eastern edges bordering dense human populations [119].

Between 1996 and 2003, 39% of gray foxes sampled for post bait-distribution monitoring were tetracycline-positive and 62% of gray foxes had RVNA ≥ 1:5 [83]. During 2012, RVNA were detected in 93% of gray foxes in West Texas after ORV [126]. Rabies cases in gray foxes decreased from 244 in 1995 (before the first ORV program) to 11 in 2008 [127]. One rabid fox was reported in 2009 and another case (in a cow) in 2013 [84]. As of this writing, there have been no additional cases of gray fox variant rabies reported in Texas [84] (Figure 6). During 2014, the ORV fox program in west-central Texas was limited to a contingency zone placed around the 2013 case [84]. From 1995 to 2014, more than 46 million RABORAL V-RG baits have been distributed in Texas rabies control and elimination campaigns in coyotes and gray foxes [128].

In the USA, rabid skunks also represent a significant public and animal health concern and pose unique challenges to disease control efforts. Skunks are a common non-target species observed during ORV programs targeting raccoons, coyotes and gray foxes. As with raccoons, several factors may affect successful ORV bait uptake in skunks, but generally skunks have demonstrated lower seroconversion rates in post-baiting monitoring compared to sympatric target species (e.g., a maximum estimate of 11% of skunks versus 32.8% of raccoons after distribution of RABORAL V-RG coated sachets at 75 baits/km^2^; [111, 129]), despite laboratory evidence that skunks mount an antibody response to ORV [88, 89] and tetracycline biomarker evidence that skunks do find and ingest vaccine-baits in the field setting [32].

Poor vaccine uptake from current bait and packaging formats may be one of the primary reasons for the low serological responses observed in skunks exposed to ORV in the field setting. Inefficient oral uptake of liquid rabies vaccines released from baits during manipulation by skunks has been observed in the captive setting [89]. While V-RG effectively protected four of six skunks from rabies virus challenge when delivered by direct instillation, administration of the same vaccine dose in the current commercial bait did not result in seroconversion or protection against challenge, presumptively due to insufficient ingestion of the liquid vaccine [89].

Compared to other ORV target species, skunks have a smaller mouth and a tendency to nibble rather than bite or gulp food, which may increase spillage of the vaccine from baits during manipulation and reduce exposure of pharyngeal tissues to the vaccine [89]. In the field setting, skunks tend to be more sedentary and have smaller home ranges than raccoons and these traits may present a barrier to vaccine uptake through reduced physical access to baits [130]. Effective application of ORV to control skunks rabies virus variants may require new baits designed for the anatomy and unique feeding behaviours of this species, as well as optimization of bait distribution strategies to increase bait discovery and uptake [31, 32].

RABORAL V-RG was conditionally licensed in the USA for raccoons in 1994 with a full license granted after proof of field effectiveness in 1997 and a coyote claim added in 2002 [30]. As of this report, RABORAL V-RG continues to be used experimentally in gray foxes and was evaluated in skunks from 2013 to 2015 in Texas.

#### Canada

As in the USA, rabies virus variants in Canada circulate in geographically limited areas and are associated with specific primary reservoir species (primarily red foxes, raccoons, skunks and bats), including recent incursions of arctic fox variant into red fox populations in Canada since the late forties [131]. Wildlife rabies is controlled in Canada similarly to the USA through reservoir population management (i.e., point infection control approaches) and ORV distribution [131]. Raccoon rabies emerged as a problem in this region as the outbreak in the USA progressed north, but incursion into Canada has been limited by aggressive management. However, as long as the north-eastern USA remains endemic for raccoon rabies the eastern international border between the USA and Canada remains a high-risk area for re-introduction of raccoon rabies virus variant. Skunks are a primary rabies reservoir in the western Manitoba, Saskatchewan, and Alberta and outbreaks are occasionally documented, but the dominant public health concern of rabies resides in the human population centres in eastern Canada [131].

From 1985 until 2004 wildlife rabies was prevented in Canada using an attenuated ERA strain (Evelyn-Rokitnicki-Abelseth [23]) of rabies virus as a vaccine to control fox rabies outbreaks primarily in Ontario [132]. The first raccoon rabies cases were detected in eastern Ontario during 1999 [133]. A point infection control strategy was employed which integrated population reduction, trap-vaccinate-release and ORV. The initial operation included concentric control zones: an inner 5-km population reduction zone, a middle 5 km trap-vaccinate-release zone and an outer 8 to 15 km ORV zone. Approximately 81 300 V-RG baits were distributed aerially at a target density of 70 baits/km^2^ in September 1999. However, 35 raccoon rabies cases were detected in the control and vaccination zones within 1 year [133]. Using this intensive approach, the raccoon rabies virus variant was contained and eliminated from Ontario in 6 years, despite the outbreak occurring in areas with relatively high raccoon densities and complex landscapes. The last raccoon rabies case of this first incursion was detected in September 2005 [31, 119].

Nearly concurrently, an outbreak of raccoon rabies also occurred on Wolfe Island, Ontario during 1999. A point infection control response was used during 2000 to control this outbreak, including application of trap-vaccinate-release during 2001 to 2002 and ORV using RABORAL V-RG baits aerially distributed at a density of 75 or 150 baits/km^2^ during 2000 and again from 2003 to 2005. No rabies cases were detected on the island since January 2000 [134].

During the response to these initial raccoon variant outbreaks in Canada, more than 3 million RABORAL V-RG baits were aerially or hand-distributed in an approximate 4000 to 9000 km^2^ area of eastern Ontario at a density of 75 or 150 baits/km^2^ from 1999 to 2006 [31]. Most of the doses (≈90%) were considered to be experimental as they were manufactured with bulk V-RG vaccine filled into baits manufactured in Canada (Ontario Slim bait, Artemis Technologies Inc., Ontario, Canada). A smaller portion of baits used in Ontario were the commercial serials of RABORAL V-RG coated sachet and FMP baits (i.e., commercially acquired products produced fully in the USA).

In post-distribution surveillance, bait uptake (as measured by tetracycline biomarker) by raccoons was significantly higher in areas baited at a density of 150 baits/km^2^ compared to 75 baits/km^2^, in areas that applied a flight line spacing of 0.75 km rather than 1.5 km and when bait distribution occurred in September rather than in June [30]. Bait acceptance was also higher in adults than juveniles at the lower bait density. Seropositivity rates in raccoons were determined using a competitive enzyme linked immunosorbent assay (ELISA) test assigned a threshold of positivity equivalent to 0.5 IU/mL ranged from 7 to 28% and from 10 to 27% in areas baited at 75 and 150/km^2^, respectively [31].

However, skunk bait acceptance and antibody response in this same study were both lower than for raccoons [31]. A palatability study testing different shapes and flavours of baits by captive striped skunks has shown that the uptake rate of the Canadian bait used to deliver V-RG was low (13 to 17%) compared to the FMP (45%) and coated sachet (42%). The waxy texture of the bait appeared to make chewing difficult for this species. In this evaluation the most effective bait format was the fish-crumble coated sachet in terms of uptake and sachet puncture. A reduced bait sachet size was proposed as a product improvement which may allow skunks to more easily puncture the vaccine container [135, 136].

Beginning in 2006 a replication-competent human adenovirus vaccine, ONRAB^®^, developed by the Ontario Ministry of Natural Resources, replaced RABORAL V-RG as the primary vaccine used in ORV programs in Ontario and Quebec to address remaining cases of raccoon rabies virus variant circulating in Canadian raccoon and skunk populations [136]. Integrative management efforts continue to be used in eastern Canada to prevent re-introduction of the raccoon rabies virus variant from endemic areas in the USA. Between 1999 and 2007, 132 cases of raccoon rabies virus variant (130 raccoons, 2 striped skunks) were reported in eastern Ontario. The last reported raccoon variant case from the 1999 incursion occurred in September 2005 [137]. Subsequently, Ontario remained raccoon variant free for 10 years before raccoon rabies was detected again in a border region near Vermont during December 2015. As of February 2017, there were six fox variant and 282 raccoon variant cases reported in Ontario associated with this re-emergence of rabies [137].

### Israel

During 1950 to 1970, golden jackals were the major reservoir of wildlife rabies in Israel [92, 138]. Rabies was mainly urban in distribution in dog populations before 1958 [138, 139]. From the mid-seventies, Israel experienced a major transition from urban dog rabies to sylvatic fox rabies, with a significant increase in cases. Foxes became the primary rabies reservoir in Israel during 1988 to 1997, accounting for 49% of all rabies cases during this period [138]. After three human rabies cases in 1996 to 1997, and an increase in animal rabies, the Veterinary Services and Nature and Parks Authority decided to initiate an ORV in wildlife [139, 140].

An ORV program was begun during the fall of 1998 in the heavily affected northern region, and then extended progressively to the majority of the country, as well as the West Bank in 2004 (in total 21 000 km^2^). During 1998 to 2004, RABORAL V-RG FMP baits were distributed in autumn and spring at a density of 14 to 19 baits/km^2^ by helicopters or light airplanes over uninhabited areas, with hand distribution in urban areas [139, 140]. During 1999 to 2004, 54.4% (43.1 to 75.7%) of jackals and foxes were tetracycline positive and 29.5% (14.0 to 66.6%) had detectable RVNA. Monitoring of bait uptake through tracking stations showed that 40 to 90% of the baits were removed during the first night. Rabies prevalence decreased sharply from 70 cases in 1998 to 3 cases in 2003 and 2004, after the first year of baiting in the rural northern Israel and the southern desert. All rabies cases detected in these areas during 2002 to 2004 were located on the border of the vaccinated areas. A decrease in rabies prevalence was observed after 2 years of ORV (a total of 4 vaccination campaigns) in the narrow urban zone located along the central coastal part of Israel adjacent to the West Bank, with 59 rabies cases in 2002, and 25 cases in 2004 [139].

**Figure 7:**
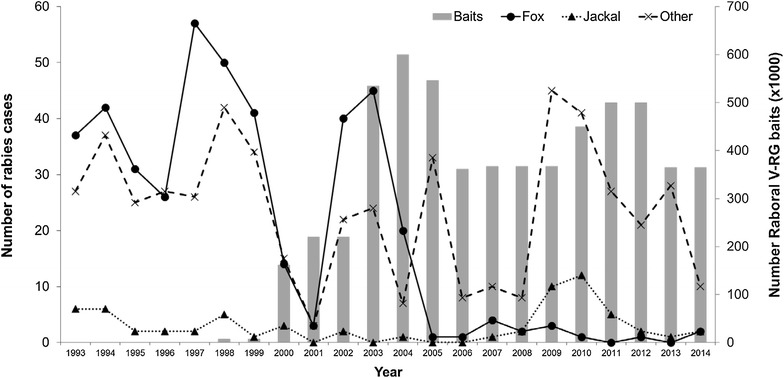
**Rabies prevalence by species and V-RG distribution volume, Israel—1993 to 2014.** Rabies prevalence in jackals, fox and other terrestrial species and V-RG bait distribution volume, in Israel—1993 to 2014 (Data source: personal communication B. Yakobson, Kimron Veterinary Institute, Israel).

ORV campaigns have been conducted annually throughout Israel since 2004. After several years of ORV, the vast majority of Israel (90%) is currently free of carnivore rabies virus variants. In 2012, two jackals and one fox were reported rabid; in 2013, there was a single rabid jackal case, and in 2014, there was a rabid jackal and a fox case [141] (Figure 7). However, while wildlife rabies remains under control, a new canine (dog) rabies virus variant, which originated from Turkey, emerged in the northern area of Golan Heights since 2004 [142]. To prevent this canine rabies virus variant from spreading, dog vaccination campaigns have been implemented in high risk regions of the country. Surveillance of rabies cases continues with spill-over cases documented in domestic animals and occasionally wildlife.

## Conclusion

Rabies epizootics in western Europe (red fox) and North America (raccoon, coyote and gray fox) urgently drove the real-time development and licensing of wildlife rabies vaccines on both continents. RABORALV-RG was a technological breakthrough vaccine in 1984, brought to fruition under circumstances supportive of international collaborations focused on protecting public health and well-being. The poxvirus vectored recombinant rabies vaccine (V-RG) provided a novel vaccine technology that met the challenges of orally vaccinating wildlife and has been used for this purpose since 1987. This recombinant live-vectored vaccine was tested extensively for environmental release to ensure the construct was stable genetically, safe in target and non-target species, and effective in preventing the spread of rabies virus in targeted animal populations. Today, the concept of immunizing wildlife with an oral vaccine is well accepted globally as an important component of a holistic rabies management program to create an additional layer of protection for domestic animals and humans and prevent the spread of rabies virus from infected reservoirs.

Nearly three decades of wildlife rabies prevention efforts in Europe and North America have demonstrated the value of oral rabies vaccines as tools for controlling outbreaks and mitigating rabies risks to humans, domestic animals and wildlife. As a tenacious ancient foe, the rabies virus will continue to adapt and perpetuate within wildlife populations. In combination with other approaches, the public health and agricultural impact of rabies can be mitigated and user some circumstances eliminated through the judicious use of effective oral rabies vaccines, such as RABORAL V-RG.

## Additional files

**Additional file 1.** **Safety testing of V-RG in non-target species.** This table contains compiled information summarizing studies of RABORAL V-RG vaccine safety in a diversity of animal species that are not the primary target for vaccination (non-target) but could be inadvertently exposed to the vaccine through environmental release.

**Additional file 2.** **Efficacy studies using V-RG in current or potential primary target species administered by various routes.** This table contains compiled information summarizing studies of RABORAL V-RG immunogenicity and efficacy in wildlife species that are the current target of label or experimental use of this vaccine or have been identified as possible future primary targets for oral vaccination to support rabies control and prevention efforts.

## Abbreviations

cDNA: complementary deoxyribonucleic acid
ELISA: enzyme linked immunosorbent assay
ERA: Elizabeth Rokitnicki Abelseth; a rabies virus strain derived from the Street Alabama Dufferin rabies virus strain
FMP: fishmeal polymer block bait
G-protein: glycoprotein
GPS: global positioning system
i.d.: intradermal
i.m.: intramuscular
ONRAB^®^: Ontario rabies vaccine bait
ORV: oral rabies vaccine/vaccination
p.o.: per os (oral installation)
PCR: polymerase chain reaction
PFU: plaque forming units
RVNA: rabies virus neutralizing antibodies
s.c.: subcutaneous injection
SAD: Street Alabama Dufferin rabies virus strain
SAD-B19: Street Alabama Dufferin B19; an attenuated rabies vaccine derived from the SAD (Street Alabama Dufferin) rabies virus strain
SAG1: SAD (Street Alabama Dufferin) Avirulent Gif 1; an avirulent mutant rabies virus vaccine
SAG2: SAD (Street Alabama Dufferin) Avirulent Gif 2; a double avirument mutant rabies virus vaccine
TCID_50_: tissue culture infective dose 50%
TK: thymidine kinase
USDA: United States Department of Agriculture
V-RG: recombinant vaccinia virus vector vaccine expressing the rabies virus glycoprotein gene
