# Supplementary material for: Oral vaccination of wildlife using a vaccinia–rabies-glycoprotein recombinant virus vaccine (RABORAL V-RG®): a global review
Source: Vet Res. 2017 Sep 22;48:57. doi: 10.1186/s13567-017-0459-9 (PMC5610451; doi:10.1186/s13567-017-0459-9)
Supplement: Supplementary file 1 — Additional file 1. Safety testing of V-RG in non-target species. This table contains compiled information summarizing studies of RABORAL V-RG vaccine safety in a diversity of animal species that are not the primary target for vaccination (non-target) but could be inadvertently exposed to the vaccine through environmental release. [file 13567_2017_459_MOESM1_ESM.docx]

Table S1. Safety testing of V-RG in non-target species

| **Taxonomy** | **Species** | **No of animals** | **Route^a^** | **Dose per animal^b^** | **Observation period (days)** | **Reference** |
| --- | --- | --- | --- | --- | --- | --- |
| **CLASS MAMMALIA** |  |  |  |  |  |  |
| **Order Marsupiala** |  |  |  |  |  |  |
| **Family Didelphidae** |  |  |  |  |  |  |
|  | Opossum *(Didelphis virginiana)* | 6 | p.o. | 10^7.0^ PFU | 30 | [145] |
| **Order Insectivora** |  |  |  |  |  |  |
| **Family Soricidae** |  |  |  |  |  |  |
|  | Short-tailed Shrew *(Blarina brevicauda)* | 1 | p.o. | 10^7.0^ PFU | 70 | [146] |
| **Order Rodentia** |  |  |  |  |  |  |
| **Family Muridae** |  |  |  |  |  |  |
|  | House Mouse *(Mus musculus)* | 12 | i.d. | 10^8.3^ PFU | 14 | [25] |
|  |  | 12 | foot pad | 10^7.7^ PFU | 14 | [25] |
|  | Wood Mouse *(Apodemus sylvaticus)* | 27 | p.o. | 10^6.3-6.5^ TCID_50_ | 28-43 | [56] |
|  | Yellow-Necked Mouse *(Apodemus flavicollis)* | 7 | p.o. | 10^6.5^ TCID_50_ | 41 | [56] |
|  | *Apodemus sp.* | 4 | p.o. | 10^6.3^ TCID_50_ | 41 | [56] |
|  | Tristram’s Jird (*Meriones tristrami*) | 10 | p.o. | 10^5.5-7.4^ TCID_50_ | 31 | [145] |
|  | Cairo Spiny Mouse (*Acomys cahirinus*) | 10 | p.o. | 10^5.5-7.4^ TCID_50_ | 31 | [145] |
| **Family Erethizontidae** |  |  |  |  |  |  |
|  | North American Porcupine *(Erethizon dorsatum)* | 3 | p.o. | 10^9.0^ PFU | 30 | [146] |
| **Family Sciuridae** |  |  |  |  |  |  |
|  | Groundhog *(Marmota monax)* | 10 | p.o. | 10^7.9^ TCID_50_ | 90 | [68] |
|  | Gray Squirrel *(Sciurus carolinensis)* | 11 | p.o. | 10^7.9^ TCID_50_ | 90 | [68] |
|  | Southern Flying Squirrel *(Glaucomys volans)* | 2 | p.o. | 10^8.0^ PFU | 90 | [146] |
| **Family Cricetidae** |  |  |  |  |  |  |
|  | Hispid Cotton rat *(Sigmodon hispidus)* | 4 | p.o. | 10^8.0^ PFU | 30 | [146] |
|  | Marsh Rice Rat *(Oryzomys palustris)* | 7 | p.o. | 10^8.0^ PFU | 60 | [146] |
|  | Syrian Hamster *(Mesocricetus auratus)* | 12 | i.m. | 10^7.0^ PFU | 30 | [147] |
|  | Field Vole *(Microtus agrestis)* | 1 | p.o. | 10^6.5^ TCID_50_ | 35 | [56] |
|  | Meadow Vole *(Microtus pennsylvanicus)* | 12 | p.o. | 10^7.9^ TCID_50_ | 90 | [68] |
|  |  | 14 | p.o. | 10^9.0^ TCID_50_ | 30 | [68] |
|  | Common Vole *(Microtus arvalis)* | 2 | p.o. | 10^6.5^ TCID_50_ | 35 | [56] |
|  | Bank Vole *(Myodes glareolus*, formerly *Clethrionomys glareolus)* | 4 | p.o. | 10^6.5^ TCID_50_ | 45 | [11] |
|  |  | 13 | p.o. | 10^6.3^ TCID_50_ | 28 | [56] |
|  | Southern Red-backed Vole *(Myodes gapperi)* | 3 | p.o. | 10^7.0^ PFU | 60-90 | [146] |
|  | European Water Vole *(Arvicola amphibius* formerly *terrestris)* | 5 | p.o. | 10^6.5^ TCID_50_ | 41 | [56] |
|  | North American Deer Mouse *(Peromyscus maniculatus)* | 10 | p.o. | 10^9.0^ PFU | 90 | [146] |
| **Family Dipodidae** |  |  |  |  |  |  |
|  | Woodland Jumping Mouse *(Napaeozapus insignis)* | 1 | p.o. | 10^7.0^ PFU | 90 | [146] |
| **Order Lagomorpha** |  |  |  |  |  |  |
| **Family Leporidae** |  |  |  |  |  |  |
|  | European Rabbit *(Oryctolagus cuniculus)* | 4 | i.d. | 10^8.3^ PFU | 14 | [25] |
|  |  | 2 | i.d. | 10^7.8^ PFU | 21 | [147] |
|  |  | 2 | i.m. | 10^7.8^ PFU | 21 | [147] |
|  |  | 2 | s.c. | 10^7.8^ PFU | 21 | [147] |
|  |  | 2 | p.o. | 10^7.8^ PFU | 21 | [147] |
|  |  | 3 | i.d. | 10^7.6^ PFU | 180 | [147] |
|  |  | 3 | i.d.^c^ | 10^7.6^ PFU | 21 | [147] |
| **Order Primates** |  |  |  |  |  |  |
| **Family Cebidae** |  |  |  |  |  |  |
|  | Squirrel monkey *(Saimiri sciureus)* | 8 | i.d. | 10^8.0^ PFU | 60 | [53] |
| **Family Hominidae** |  |  |  |  |  |  |
|  | Chimpanzee *(Pan troglodytes)* | 8 | p.o. | 10^7.2^ PFU | 60 | [53] |
|  |  | 8 | p.o. | 10^9.0^ PFU | 60 | [53] |
|  |  | 3 | p.o. | 10^9.0^ PFU | 60 | [53] |
| **Order Carnivora^e^** |  |  |  |  |  |  |
| **Family Canidae** |  |  |  |  |  |  |
|  | Domestic Dog *(Canis familiaris)* | 4 | p.o. | 10^8.6^ PFU | 69 | [64] |
|  |  | 4 | p.o. | 10^9.6^ PFU | 69 | [64] |
|  |  | 3 | s.c. | 10^4.6^ TCID_50_ | 69 | [148] |
|  |  | 3 | s.c. | 10^6.6^ TCID_50_ | 69 | [148] |
|  |  | 3 | s.c. | 10^8.6^ TCID_50_ | 69 | [148] |
|  |  | 18 | p.o. | 10^8.4^ TCID_50_ | 120 | [91] |
| **Family Felidae** |  |  |  |  |  |  |
|  | Bobcat *(Lynx rufus)* | 3 | p.o. | 10^9.0^ PFU | 30 | [146] |
|  | Domestic Cat *(Felis domesticus)* | 4 | p.o. | 10^8.0^ PFU | 115 | [64] |
|  |  | 3 | s.c. | 10^4.0^ TCID_50_ | 52 | [148] |
|  |  | 3 | s.c. | 10^6.0^ TCID_50_ | 52 | [148] |
|  |  | 3 | s.c. | 10^8.0^ TCID_50_ | 52 | [148] |
| **Family Mustelidae** |  |  |  |  |  |  |
|  | Ferret *(Mustela putorius)* | 2 | p.o. | 10^8.0^ TCID_50_ | 28 | [148] |
|  |  | 2 | p.o. | 10^9.0^ TCID_50_ | 28 | [148] |
|  | Mink *(Neovison vison)* | 7 | p.o., i.d. | 10^7.7^ PFU | 180 | [146] |
|  | North American River Otter *(Lontra canadensis)* | 3 | p.o. | 10^9.0^ PFU | 30 | [146] |
| **Family Ursidae** |  |  |  |  |  |  |
|  | American Black Bear *(Ursus americanus)* | 3 | p.o. | 10^8.8^ PFU | 30 | [146] |
| **Order Artiodactyla** |  |  |  |  |  |  |
| **Family Bovidae** |  |  |  |  |  |  |
|  | Cattle *(Bos taurus)* | 2 | i.d. | 10^8.3^ TCID_50_ | 35 | [148] |
|  |  | 1 | i.m. | 10^8.0^ TCID_50_ | 35 | [148] |
|  |  | 1 | s.c. | 10^8.0^ TCID_50_ | 35 | [148] |
|  |  | 10 | s.c. | 10^8.0^ PFU | 30 | [145] |
|  |  | 10 | i.d. | 10^8.0^ PFU | 30 | [145] |
|  | Sheep *(Ovis ovis)* | 4 | p.o. | 10^7.0^ PFU | 30 | [149] |
| **Family Suidae** |  |  |  |  |  |  |
|  | Wild Boar *(Sus scrofa)* | 4 | p.o. | 10^8.3^ TCID_50_ | 88 | [56] |
| **Family Cervidae** |  |  |  |  |  |  |
|  | White-Tailed Deer *(Odocoileus virginianus)* | 4 | p.o. | 10^9.0^ PFU | 30 | [146] |
|  |  |  |  |  |  |  |
| **CLASS AVES** |  |  |  |  |  |  |
| **Order Accipitriformes** |  |  |  |  |  |  |
| **Family Accipitridae** |  |  |  |  |  |  |
|  | Red-tailed Hawk *(Buteo jamaicensis)* | 6 | p.o. | 10^8.0^ TCID_50_ | 30 | [68] |
|  | Common Buzzard *(Buteo buteo)* | 8 | p.o. | 10^8.0^ TCID_50_ | 30-45 | [56] |
| **Order Falconiformes** |  |  |  |  |  |  |
| **Family Falconidae** |  |  |  |  |  |  |
|  | Common Kestrel *(Falco tinnunculus)* | 4 | p.o. | 10^8.0^ TCID_50_ | 30-45 | [56] |
| **Order Charadriiformes** |  |  |  |  |  |  |
| **Family Laridae** |  |  |  |  |  |  |
|  | Ring-billed Gull *(Larus delawarensis)* | 2 | p.o. | 10^7.9-8.1^ TCID_50_ | 90 | [68] |
| **Order Strigiformes** |  |  |  |  |  |  |
| **Family Strigidae** |  |  |  |  |  |  |
|  | Great Horned Owl *(Bubo virginianus)* | 8 | p.o. | 10^8.0^ TCID_50_ | 30 | [68] |
| **Order Passeriformes** |  |  |  |  |  |  |
| **Family Corvidae** |  |  |  |  |  |  |
|  | Common Magpie *(Pica pica)* | 7 | p.o. | 10^8.0^ TCID_50_ | 28 | [56] |
|  | Eurasian Jay *(Garrulus glandarius)* | 2 | p.o. | 10^8.0^ TCID_50_ | 28 | [56] |
|  | Carrion Crow *(Corvus corone)* | 17 | p.o. | 10^8.0^ TCID_50_ | 28 | [56] |

a. i.d. = intradermal; i.m. = intramuscular; p.o. = per os (oral); s.c. = subcutaneous

b. TCID_50_: median tissue culture infectious doses; PFU: plaque forming units

c. second (booster) dose administered 6 months after primary dose

d. second (booster) dose administered 2 months after primary dose

e. see Table 1 for other members of order Carnivora that are current or potential primary targets for oral rabies vaccination.
