# Supplementary material for: Oral vaccination of wildlife using a vaccinia–rabies-glycoprotein recombinant virus vaccine (RABORAL V-RG®): a global review
Source: Vet Res. 2017 Sep 22;48:57. doi: 10.1186/s13567-017-0459-9 (PMC5610451; doi:10.1186/s13567-017-0459-9)
Supplement: Supplementary file 2 — Additional file 2. Efficacy studies using V-RG in current or potential primary target species administered by various routes. This table contains compiled information summarizing studies of RABORAL V-RG immunogenicity and efficacy in wildlife species that are the current target of label or experimental use of this vaccine or have been identified as possible future primary targets for oral vaccination to support rabies control and prevention efforts. [file 13567_2017_459_MOESM2_ESM.docx]

Table S2. Efficacy studies using V-RG in current or potential primary target species administered by various routes

| **Species** | **No of animals** | **Route^a^** | **Dose per animal^b^** | **Challenge after vaccination** | | **Seroconversion** | **Survival after challenge** | | **Rabies virus positive brains** | **Reference** |
| --- | --- | --- | --- | --- | --- | --- | --- | --- | --- | --- |
| Red fox | 2 | i.d. | 10^8.0^ PFU | 28 days | | 2/2 | 2/2 | |  | [54] |
| *(Vulpes vulpes)* | 2 | s.c. | 10^8.0^ PFU | 28 days | | 1/2 | 2/2 | |  | [54] |
|  | 4 | p.o. scarified | 10^8.0^ PFU | 28 days | | 4/4 | 4/4 | |  | [54] |
|  | 4 | p.o. | 10^4.0^ PFU | 28 days | | 1/4 | 1/4 | |  | [54] |
|  | 4 | p.o. | 10^6.0^ PFU | 28 days | | 2/4 | 2/4 | |  | [54] |
|  | 4 | p.o. | 10^8.0^ PFU | 28 days | | 4/4 | 4/4 | |  | [54] |
|  | 5 | p.o. in bait | 10^8.0^ PFU/bait | 28 days | | 5/5 | 4/5 | |  | [54] |
|  | 6 | control | - | 28 days | | 0/6 | 0/6 | |  | [54] |
|  | 8 | p.o. | 10^9.1^ TCID_50_ | 90 days | | 8/8 | 8/8 | | 0/8 | [63] |
|  | 6 | intragastric | 10^9.1^ TCID_50_ | 90 days | | 3/6 | 3/6 | | 3/6 | [63] |
|  | 2 | i.d. scarified | 10^7.9^ TCID_50_ | 90 days | | 2/2 | 2/2 | | 0/2 | [63] |
|  | 8 | control | - | 90 days | |  | 0/8 | | 8/8 | [63] |
|  | 4 | p.o. | 10^6.0^ PFU | 30 days | | 1/4 | 2/4 | |  | [64] |
|  | 4 | p.o. | 10^7.0^ PFU | 30 days | | 3/4 | 4/4 | |  | [64] |
|  | 4 | p.o. | 10^8.0^ PFU | 30 days | | 3/4 | 4/4 | |  | [64] |
|  | 4^c^ | control | - |  | | 1/4 | 1/4 | |  | [64] |
|  | 4 | p.o. (lyophilised) | 10^8.0^ PFU | 30 days | | 0/4 | 3/4 | |  | [64] |
|  | 5 | p.o. in bait | 10^8.0^ PFU/bait | 30 days | | 5/5 | 4/5 | |  | [64] |
|  | 4 | control | - | 30 days | |  | 0/4 | |  | [64] |
|  | 6 | p.o. | 10^8.0^ PFU | 1 month | | 6/6 | 6/6 | |  | [64] |
|  | 2 | control | - | 1 month | |  | 2/2 | |  | [64] |
|  | 4 | p.o. | 10^8.0^ PFU | 6 months | | 3/4 | 2/4 | |  | [64] |
|  | 2 | control | - | 6 months | |  | 0/2 | |  | [64] |
|  | 4 | p.o. | 10^8.0^ PFU | 12 months | | 4/4 | 4/4 | |  | [64] |
|  | 2 | control | - | 12 months | |  | 0/2 | |  | [64] |
|  | 4 | p.o. | 10^8.0^ PFU | 18 months | | 4/4 | 4/4 | |  | [64] |
|  | 4 | control | - | 18 months | |  | 0/2 | |  | [64] |
|  | 4 | p.o. | 10^4.0^ TCID_50_ | 28 days | |  | 0/4 | | 4/4 | unpublished data^d^ |
|  | 5 | p.o. | 10^6.0^ TCID_50_ | 28 days | |  | 5/5 | | 0/5 | unpublished data^d^ |
|  | 5 | p.o. | 10^7.0^ TCID_50_ | 28 days | |  | 4/5 | | 1/5 | unpublished data^d^ |
|  | 5 | p.o. | 10^8.0^ TCID_50_ | 28 days | |  | 5/5 | | 0/5 | unpublished data^d^ |
|  | 3 | control | - | 28 days | |  | 0/3 | | 3/3 | unpublished data^d^ |
|  | 8 | p.o., 2 doses 121 days apart in bait | 10^7.6-9.5^ TCID_50_/bait | 387 days (176 days after second dose) | | 5/8 | 7/8 | | 1/5 | [144] |
|  | 2 | control | - | 387 days | | 0/2 | 0/2 | | 2/2 | [144] |
| Red fox (silver var.) | 8 | p.o. | 10^8.4^ TCID_50_ | 195 days | | 8/8 | 8/8 | | 0/8 | [81] |
| (*V. vulpes*) | 8 | p.o., 2 doses 35 days apart | 10^8.4^ TCID_50_ | 195 days | | 8/8 | 8//8 | | 0/8 | [81] |
|  | 4 | control | - | 195 days | | 0/4 | 0/4 | | 4/4 | [81] |
| Red fox kits | 5 | p.o. | 10^7.2^ PFU | 33 days | | 5/5 | 5/5 | |  | [55] |
| *(V. vulpes* silver var.) | 4 | p.o. | 10^7.2^ PFU | 180 days | | 4/4 | 4/4 | |  | [55] |
|  | 3 | p.o. | 10^7.2^ PFU | 360 days | | 2/3 | 2/3 | | 1/1 | [55] |
|  | 4 | control | - |  | |  | 0/4 | | 4/4 | [55] |
| Red fox (silver or | 10 | p.o. in square bait | 10^8.4^ TCID_50_/bait | 91 days | | 8/10 | 8/10 | | 2/10 | [91] |
| golden var.) | 10 | control (placebo bait) | - | 91 days | | 1/10 | 0/10 | | 10/10 | [91] |
| (*V. vulpes*) | 10 | p.o. in rectangular bait | 10^8.3^ TCID_50_/bait | 91 days | | 6/10 | 7/10 | | 3/10 | [91] |
|  | 6 | control (no bait) | - | 91 days | | 0/6 | 0/6 | | 6/6 | [91] |
| Red fox kits | 6 | p.o. (1 bait on day 1) | 10^8.0^ TCID_50_/bait | 30 days | | 5/6 | 5/6 | | 1/1 | [65] |
| (*V. vulpes*) | 6 | p.o. (1 bait on days 1 and 2) | 10^8.0^ TCID_50_/bait | 30 days | | 5/6 | 6/6 | |  | [65] |
|  | 6 | p.o. (1 bait on days 1, 2 and 3) | 10^8.0^ TCID_50_/bait | 30 days | | 5/6 | 5/6 | | 1/1 | [65] |
|  | 4 | controls | - | 30 days | | 0/4 | 0/4 | | 4/4 | [65] |
| Red fox kits | 16 (born to vaccinated vixens) | p.o. | 10^8.0-8.4^ TCID_50_  at 30 days of age | 5 months of age | |  | 16/16 | | 0/16 | [80] |
| (*V. vulpes* silver var.) | 19 (born to unvaccinated vixens) | p.o. | 10^8.0-8.4^ TCID_50_  at 30 days of age | 5 months of age | |  | 19/19 | | 0/19 | [80] |
|  | 17 (born to vaccinated vixens) | p.o. | 10^8.0-8.4^ TCID_50_  at 90 days of age | 5 months of age | |  | 17/17 | | 0/17 | [80] |
|  | 13 (born to unvaccinated vixens) | p.o. | 10^8.0-8.4^ TCID_50_  at 90 days of age | 5 months of age | |  | 13/13 | | 0/13 | [80] |
|  | 28 | controls | - | 5 months of age | |  | 1/28 | | 27/28 | [80] |
| Arctic fox  *(Vulpes lagopus)* | 8 | p.o. | 10^8.0^ PFU | 16 weeks | | 8/8 | 8/8 | | 0/8 | [82] |
|  | 3 | controls | - | 16 weeks | | 0/3 | 1/3 | | 2/3 | [82] |
| Raccoon  *(Procyon lotor)* | 8 | p.o. (sponge baits) | 10^8.0^ PFU/bait | 28 days | | 8/8 | 8/8 | |  | [16] |
|  | 1 | p.o. (oral infusion) | 10^6.0^ PFU | 28 days | | 1/1 | 0/1 | | 1/1 | [16] |
|  | 1 | p.o. (oral infusion) | 10^8.0^ PFU | 28 days | | 1/1 | 1/1 | |  | [16] |
|  | 6 | controls (placebo bait) | - | 28 days | | 0/6 | 0/6 | | 6/6 | [16] |
|  | 10 | p.o. (sponge baits) | 10^8.0^ PFU | 205 days | | 10/10 | 8/10 | | 2/2 | [16] |
|  | 7 | controls (placebo bait) | - | 205 days | |  | 1/7 | | 6/6 | [16] |
|  | 3 | i.d. | 10^7.0^ PFU | 28 days | | 3/3 | 3/3 | |  | [52] |
|  | 3 | i.m. | 10^7.8^ PFU | 63 days | | 3/3 | 2/3 | |  | [52] |
|  | 6 | p.o. | 10^8.0^ PFU | 28 days | | 6/6 | 6/6 | |  | [52] |
|  | 1 | p.o. (day 0 and booster on day 42) | 10^7.8^ PFU | 63 days | | 1/1 | 1/1 | |  | [52] |
|  | 2 | p.o. (buccal scarification, day 0 and booster on day 42 | 10^7.8^ PFU | 63 days | | 2/2 | 1/2 | |  | [52] |
|  | 7 | p.o. (bait) | 10^8.0^ PFU/bait | 6 months | | not determined | 7/7 | | 0/7 | [66] |
|  | 8 | controls | - | 6 months | | not determined | 0/8 | | 8/8 | [66] |
| Coyote | 4 | p.o. | 10^7.4^ TCID_50_ | 49 days | | 4/4 | 4/4 | |  | unpublished data^e^ |
| *(Canis latrans)* | 8 | p.o. | 10^8.3^ TCID_50_ | 49 days | | 8/8 | 8/8 | |  | unpublished data^e^ |
|  | 6 | controls | - | 49 days | | 0/6 | 0/6 | |  | unpublished data^e^ |
| Striped Skunk | 8 | p.o. (bait) | 10^9^ PFU/bait | 90 days | | 6/7 | 5/7 | | 2/7 | [88] |
| *(Mephitis mephitis)* | 8 | intraduodenal | 10^9^ PFU | 90 days | | 5/8 | 4/8 | | 4/8 | [88] |
|  | 4 | i.m. | 10^8.3^ PFU | 90 days | | 4/4 | 3/4 | | 1/4 | [88] |
|  | 6 | i.d. | 10^8.3^ PFU | 90 days | | 6/6 | 5/6 | | 1/6 | [88] |
|  | 8 | controls | - |  | |  | 0/8 | | 8/8 | [88] |
|  | 6 | p.o. | 10^7.7^ TCID_50_ | 116 days | | 4/6 | 4/6 | | 2/6 | [89] |
|  | 5 | p.o. (1 coated sachet) | 10^7.7^ TCID_50_/bait | 116 days | | 0/5 | 1/5 | | 4/5 | [89] |
|  | 5 | p.o. (3 coated sachets) | 10^7.7^ TCID_50_/bait | 116 days | | 0/5 | 0/5 | | 5/5 | [89] |
|  | 6 | controls | - |  | | - | 0/6 | | 6/6 | [89] |
| Raccoon dog | 10 | p.o. (square bait) | 10^8.8^ TCID_50_/bait | 124 days | | 10/10 | 10/10 | | 0/10 | [91] |
| *(Nyctereutes* | 10 | p.o. (rectangular bait) | 10^9.0^ TCID_50_/bait | 124 days | | 10/10 | 10/10 | | 0/10 | [91] |
| *procyonoides)* | 9 | controls | - | 124 days | | 0/9 | 0/9 | | 9/9 | [91] |
| Golden Jackal | 9 | p.o. (bait) | 10^8.0^ TCID_50_/bait | 160 days | | 4/9 | 7/9 | | - | [92] |
| *(Canis aureus)* | 10 | controls | - | 160 days | | - | 0/10 | | - | [92] |
|  | 8 | p.o., 2 doses 121 days apart in bait | 10^7.6-9.5^ TCID_50_/bait | 387 days (176 days after second dose) | 3/8 | | | 8/8 | 0/8 | [150] |
|  | 3 | p.o. | 10^7.6-9.5^ TCID_50_ | 207 | | 3/3 | 3/3 | | 0/3 | [150] |
|  | 2 | Control | - | 387 days | | 0/2 | 2/2 | | 0/2 | [150] |
| Small Asian Mongoose | 5 | p.o. | 10^8.0^ TCID_50_ | 28 days | | 0/5 | 1/5 | | 4/5 | [93] |
| *(Herpestes javanicus)* | 7 | controls | Medium | 28 days | | 0/5 | 0/5 | | 5/5 | [93] |
| European Badger  *(Meles meles)* | 6 | p.o. | 10^8.3^ TCID_50_ | 45 days | | 2/6 | 2/5 | | 3/5 | [56] |
|  | 4 | controls | - | 45 days | | 0/4 | 0/4 | | 4/4 | [56] |
| Vampire bat | 8 | p.o. | 10^8.0^ TCID_50_ | 120 days | | 1/8 | 1/8 | | 7/7 | [98] |
|  | 10 | p.o. | 10^8.0^ TCID_50_ | 30 days | | 5/10 | 8/10 | | 2/2 | [98] |
| *(Desmodus rotundus)* | 10 | controls | - |  | | 0/10 | 2/10 | | 8/8 | [98] |
|  | 9 | p.o. | 10^8.0^ TCID_50_ | 90 days | | 4/9 | 3/9 | | 6/6 | [98] |
|  | 10 | controls | - |  | | 0/10 | 0/10 | | 10/10 | [98] |
|  | 10 | p.o. | 10^8.0^ TCID_50_ | 18 days | | 0/10 | 6/10 | | 4/4 | [98] |
|  | 9 | p.o. | 10^8.0^ TCID_50_ | day of vaccination | | 0/9 | 0/9 | | 9/9 | [98] |
|  | 10 | p.o. | 10^8.0^ TCID_50_ | 5 days pre-vaccination | | 0/10 | 1/10 | | 9/9 | [98] |
|  | 10 | control | - | - | | 0/10 | 1/10 | | 9/9 | [98] |
|  | 8 | i.m. | 10^7.4^ TCID_50_ | 31 days | | 8/8 | 8/8 | | 0/8 | [99] |
|  | 8 | i.d. scarified | 10^7.0^ TCID_50_ | 31 days | | 8/8 | 8/8 | | 0/8 | [99] |
|  | 8 | p.o. | 10^8.0^ TCID_50_ | 31 days | | 3/8 | 8/8 | | 0/8 | [99] |
|  | 8 | aerosol | 10^7.4^ TCID_50_ | 31 days | | 4/8 | 7/8 | | 1/8 | [99] |
|  | 10 | control | saline injected i.m. | 31 days | | 0/10 | 1/10 | | 9/10 | [99] |
|  | 14 | p.o. | 0.7 ml (unknown dose) | 25 days | | 14/14 | 12/14 | | 2/14 | [100] |
|  | 21 | contact | Unknown | 25 days | | 16/19 | 17/21 | | 4/21 | [100] |
|  | 7 | control | - | 25 days | | 0/7 | 3/7 | | 4/7 | [100] |

a. i.d. = intradermal; i.m. = intramuscular; p.o. = per os (oral); s.c. = subcutaneous; contact = indirect exposure to vaccine by contact with vaccinated animals; control = no vaccine given

b. TCID50: median tissue culture infectious doses; PFU: plaque forming units

c. One control was bitten by a dog that was vaccinated few minutes before the bite was delivered.

d. Sanofi-Pasteur unpublished data; European registration dossier JL/JL/SEB.93/D001

e. unpublished data; US registration dossier, VRG 94/069
